# MET as resistance factor for afatinib therapy and motility driver in gastric cancer cells

**DOI:** 10.1371/journal.pone.0223225

**Published:** 2019-09-26

**Authors:** Karolin Ebert, Julian Mattes, Thomas Kunzke, Gwen Zwingenberger, Birgit Luber

**Affiliations:** 1 Technische Universität München, Fakultät für Medizin, Klinikum rechts der Isar, Institut für Allgemeine Pathologie und Pathologische Anatomie, Trogerstr, München, Germany; 2 MATTES Medical Imaging GmbH, Softwarepark, Hagenberg, Austria; National University Singapore Yong Loo Lin School of Medicine, SINGAPORE

## Abstract

The therapeutic options for advanced gastric cancer are still limited. Several drugs targeting the epidermal growth factor receptor family have been developed. So far, the HER2 antibody trastuzumab is the only drug targeting the HER-family that is available to gastric cancer patients. The pan-HER inhibitor afatinib is currently investigated in clinical trials and shows promising results in cell culture experiments and patient-derived xenograft (PDX) models. However, some cell lines do not respond to afatinib treatment. The determination of resistance factors in these cell lines can help to find the best treatment option for gastric cancer patients. In this study, we analyzed the role of MET as a resistance factor for afatinib therapy in a gastric cancer cell line. MET expression in afatinib-resistant *MET*-amplified Hs746T cells was reduced by means of siRNA transfection. The effects of MET knockdown on signal transduction, cell proliferation and motility were examined. In addition to the manual assessment of cell motility, a computational motility analysis involving parameters such as (approximate) average speed, displacement entropy or radial effectiveness was realized. Moreover, the impact of afatinib was compared between MET knockdown cells and control cells. MET knockdown in Hs746T cells resulted in impaired signal transduction and reduced cell proliferation and motility. Moreover, the afatinib resistance of Hs746T cells was reversed after MET knockdown. Therefore, the amplification of *MET* is confirmed as a resistance factor in gastric cancer cells. Whether MET is a useful resistance marker for afatinib therapy or other HER-targeting drugs in patients should be investigated in clinical trials.

## 1. Introduction

Gastric cancer, an important malignancy worldwide, is the fifth most frequently diagnosed cancer and the third leading cause of cancer death [1]. Although advances in therapy are made, the prognosis for the local and advanced stages of the disease is still poor [2]. In addition to conventional cytotoxic chemotherapy, there are new therapeutic options that have HER2 as a therapeutic target or activate the immune response, to give a few examples [3].

To date, the HER2 antibody trastuzumab is the only anti-HER therapeutic which is available to patients with advanced gastric cancer. Since trastuzumab is only approved for HER2-positive gastric cancers (6–30%) and approximately 50% of HER2-positive cancers are resistant to trastuzumab treatment, there is an urgent need for alternative therapies (reviewed by [4]). The effects of the pan-HER inhibitor afatinib on tumor growth in HER2-positive esophagogastric cancers not responding to trastuzumab are currently examined in a phase II clinical trial (NCT01522768).

We previously compared the effects of trastuzumab and afatinib on kinase activity in gastric cancer cell lines. Besides inhibiting the phosphorylation of HER2, EGFR and HER3, the tyrosine kinase inhibitor afatinib also had strong effects on downstream kinases MAPK1/2, AKT 1/2/3, PRAS40 and WNK1 in NCI-N87 cells. Moreover, cell proliferation was markedly reduced after afatinib treatment. By showing afatinib resistance in the *MET*-amplified cell line Hs746T, we suggested MET as resistance factor for afatinib therapy [5].

The effects of afatinib were also investigated in mouse xenograft and patient-derived xenograft (PDX) models. Afatinib treatment resulted in a dramatic regression of tumor volume in a NCI-N87 mouse xenograft model [6, 7]. In patient-derived xenograft models, afatinib inhibited tumor growth in three cases with EGFR overexpression, *EGFR* amplification or *HER2* amplification, respectively [8].

Taken together, data from cell culture and xenograft models reveal afatinib as a promising candidate for gastric cancer therapy. However, the influence of response and resistance factors on therapy outcome needs further evaluation and should be considered carefully.

The hepatocyte growth factor receptor (MET) pathway plays an important role in the regulation of growth, survival and invasiveness of gastric cancer [9, 10]. Aberrant activation of the MET signaling pathway has been associated with poor clinical outcomes, suggesting the therapeutic potential of MET [10, 11]. Different antibodies targeting MET or its ligand HGF, and tyrosine kinase inhibitors targeting MET are investigated in clinical trials with gastric cancer patients. The anti-HGF antibody rilotumumab did not improve the clinical outcome in MET-positive advanced gastric cancer or gastroesophageal junction (GEJ) cancer in a phase III study (RILOMET-1) [12]. The MET antibody onartuzumab failed to improve outcome in patients with HER2-negative and MET-positive advanced gastric or GEJ cancer [13]. A phase I study showed promising results for the MET antibody ABT-700 as monotherapy in *MET*-amplified advanced solid tumors. A partial response was demonstrated in 3 out of 5 patients with *MET-*amplified tumors (ovarian, gastric, esophageal), whereas the 36 patients with tumors without *MET* amplification did not respond [14]. In a phase Ib/II study, patients with *MET*-amplified gastric, esophageal or GEJ cancer responded to tyrosine kinase inhibitor AMG-337 [15]. The phase II study investigating AMG-337 in *MET*-amplified gastric/esophageal carcinoma or other solid tumors was terminated (NCT02016534). However, another phase II study currently investigates AMG-337 in advanced solid tumors that overexpress MET or bear *MET* exon 14 skipping (NCT03147976).

In this study, we investigated the role of MET as a resistance factor for afatinib therapy in the gastric cancer cell line Hs746T by means of MET knockdown. The effects of MET knockdown on signal transduction and its phenotypic effects on cell proliferation and cell motility were considered. We were able to show at the molecular and phenotypic level that it is possible to restore a therapeutic response to afatinib therapy by downregulation of MET.

## 2. Materials and methods

### 2.1 Cell culture

The gastric cancer cell line Hs746T (ATCC) was cultured at 37°C in humidified atmosphere with 5% CO_2_. Cells were grown in Dulbecco’s Modified Eagle Medium with GlutaMAX (Thermo Fisher Scientific) with 0.5% penicillin/streptomycin (Thermo Fisher Scientific) and 10% Sera Plus (Pan Biotech). The absence of mycoplasma was tested as described elsewhere [5].

### 2.2 Transfection with siRNA

Hs746T cells were plated one day before transfection with a density of 1.7 x 10^4^ cells/cm^2^. Two hours before transfection, the medium was replaced by antibiotic free medium. Cells were transfected with a pool of 4 siRNA oligomers (5.7 pmol/cm^2^) against MET (Flexi Tube Gene Solution, Qiagen) and 0.57 μl/cm^2^ Lipofectamin^®^2000 (Thermo Fisher Scientific) according to the manufacturer’s instruction. As negative control, cells were transfected with equal amounts of All Star Negative Control siRNA (Qiagen). All Star Negative Control siRNA AF488 (Qiagen) was used to determine the transfection efficiency. The transfection was stopped by medium replacement after 24 h. Cells were then plated for proliferation assay, motility analysis and generation of protein lysates.

### 2.3 Western blot analysis

Western blot analyses were performed according to a standard protocol described earlier [16, 17]. One day after transfection, cells were harvested and plated. The following day they were treated for 20 minutes with 0.5 μM afatinib (Biozol) or 0.05% DMSO (solvent control). 15–25 μg of protein was loaded for each lane. Antibodies against MET D1C2 (#8198, 1:1000 in 5% BSA), pMET Y1234/1235 (#3077, 1:1000 in 5% BSA), EGFR (#2232, 1:1000 in 5% skimmed milk), pMAPK T202/Y204 (#9101, 1:2000 in 5% skimmed milk), pAKT S473 (#4060, 1:2000 in 5% BSA), anti-rabbit IgG (#7074, 1:2000 in TBST) (all Cell Signaling Technology), pEGFR Y1068 (#44788G, 1:2000 in 5% skimmed milk, Thermo Fisher Scientific), β-Actin (#A1978, 1:5000 in 5% BSA, Sigma-Aldrich) and anti-mouse IgG (#NA931, 1:10000 in 5% skimmed milk, GE Healthcare, distributed by VWR) were used. The proteins pMET and pMAPK as well as EGFR and pAKT were analyzed on the same membrane.

### 2.4 WST-1 cell proliferation assay

Cell proliferation was analyzed using WST-1 cell proliferation assay (Roche Diagnostics) as described earlier [5]. Hs746T cells were plated at a density of 4.5 x 10^3^ cells/cm^2^ and were treated with 0.5 μM afatinib or 0.05% DMSO (solvent control) for 72 h.

### 2.5 Time-lapse microscopy

Glass-bottom culture dishes (MatTek Corporation) were coated with 100 μg/ml collagen type I (BD Bioscience, distributed by VWR) for 30 minutes at 37°C. One day after transfection, 1.5 x 10^5^ cells/dish were plated onto the coated plate. In order to determine the percentage of motile cells and the average speed, time-lapse microscopy was carried out as described earlier [18, 19].

### 2.6 Computational cellular motility analysis

For each image frame of a video and for each cell having its nucleus entirely present on the frame a point in the center of the nucleus is placed and its position (x, y) is recorded together with the number t of the frame on which the point is placed, leading to a value (x, y, t) for each frame and cell.

This procedure is carried out based on an algorithm ([20]; software framework: MATTES Medical Imaging GmbH) which proposes for a given (x, y, t) position either an improved position (x', y', t) on the same frame or a position (x'', y'', t+1) on the next frame of the video's image sequence. For each cell at least one initial point for starting this algorithm has to be placed either manually or by another algorithm (for instance [21]). We used manual initialization.

Those cells–and corresponding (x, y, t)-sequences (with 1 ≤ t ≤ N, where N is the last frame of the image sequence)–have been selected which did not undergo division and for which the (x, y, t)-values are available for each image frame t. For a given (x, y, t)-sequence in a first step a sequence of displacement vectors is computed (u, v, t) = (x'-x, y'-y, t) where (x, y, t) and (x', y', t+1) correspond to the cell's position in two successive frames and the displacement length is $dt=\sqrt{u^{2}+v^{2}}$. From the sequence of displacement lengths dt different motion parameters, in particular, the approximate average speed, the displacement entropy and the radial effectiveness are computed.

The approximate average speed is defined as the approximate trajectory length divided by the total elapsed time of the video sequence. To compute the approximate trajectory length for a given minimum displacement length d_min_ (5.0 times the pixel length are chosen), sub-sequences of displacements (u_0_, v_0_, t), (u_1_, v_1_, t+1), … (u_k_, v_k_, t+k), relating the points (x_0_, y_0_, t), (x_1_, y_1_, t+1), …, (x_k+1_, y_k+1_, t+k+1), are replaced by the displacement (x_k+1_—x_0_, y_k+1_—y_0_, t+k) with length d_approx_(t,k) for the first k for which d_approx_(t,k) ≥ d_min_ (or for k = N-t-1 if for all k d_approx_(t,k) < d_min_).

**Fig 1:**
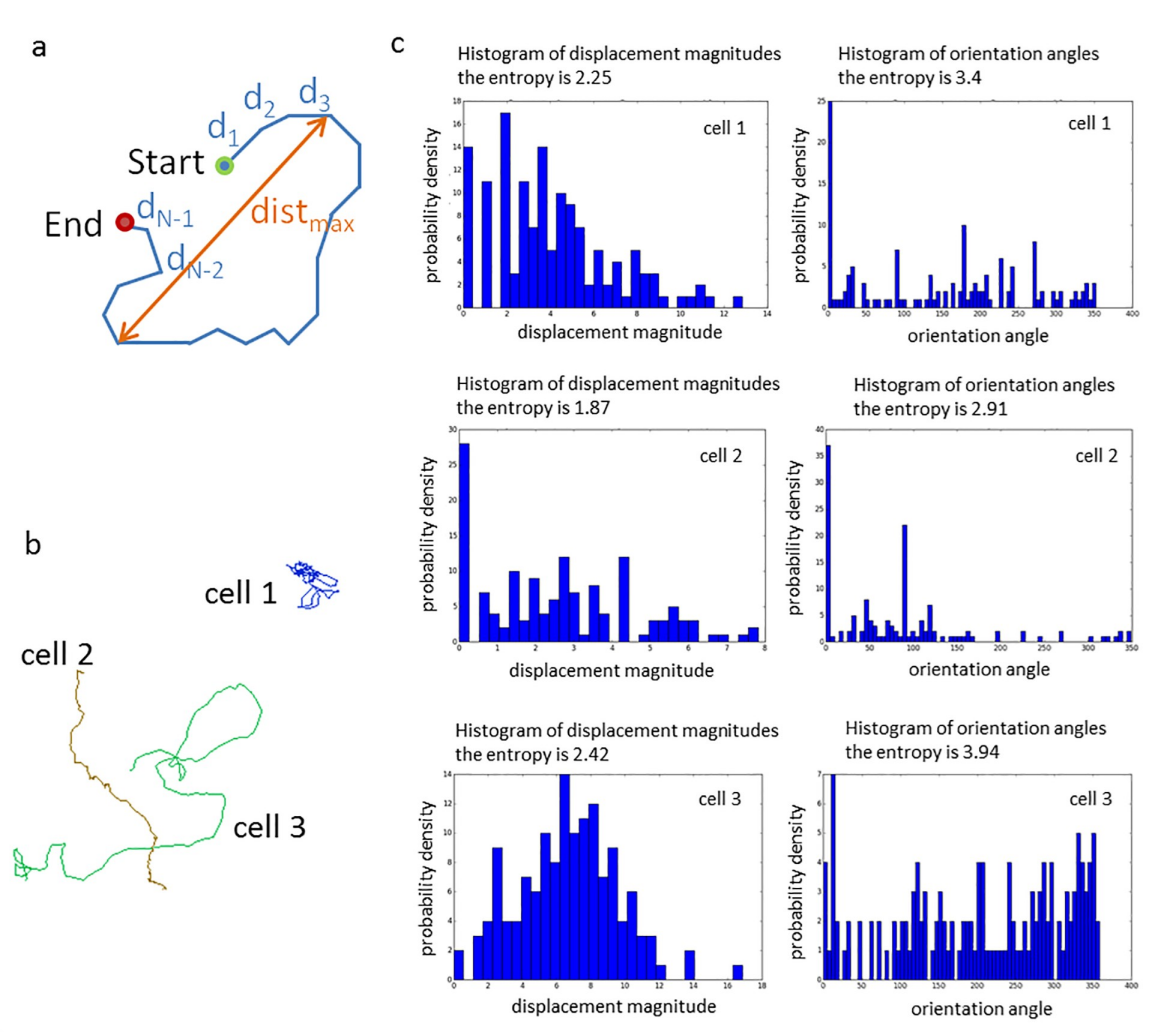
Illustration of motility parameters. (a) Schematic representation of the definition of the radial effectiveness e_r_ which is computed as the quotient e_r_ = (d_0_ + d_1_ + … + d_N-2_ + d_N-1_) / dist_max_. (b) 3 selected trajectories of cell 1, cell 2 and cell 3 colour coded by their respective e_r_ values (e_r_ = 0.11, 0.61 and 0.33 for trajectories 1, 2 and 3, respectively). (c) For each trajectory depicted in (b) the histograms of their displacement lengths/magnitudes and of their orientation angles are shown. A more concentrated histogram leads to a smaller entropy value. Thus, for trajectory 2 the smallest entropy values are obtained (1.87 and 2.91) and for trajectory 3 the highest ones (2.42 and 3.94).

The displacement entropy consists in a magnitude and in an orientation entropy where the former one is the entropy value computed for the histogram of displacement lengths dt [22]. One pixel length is taken as length of each histogram bin. The frequency hi of appearances of displacement lengths in bin i is defined as the number n_i_ of displacements dt (in pixel length) with i ≤ dt < i + 1 divided by the total number N-1 of displacements of the considered trajectory, hence h_i_ = n_i_ / (N-1). The entropy value is then calculated as the sum of all terms -h_i_*log(h_i_) for all histogram bins i. The natural logarithm log = ln to the base e (Euler's number) is taken. In the same way the orientation entropy is defined by replacing the displacement lengths by the angles of the displacement vectors with respect to the x axis. The parameters displacement entropy and orientation entropy are illustrated in Fig 1B and 1C.

The displacement effectiveness is defined as the ratio of the direct distance between the first and last point of the trajectory divided by the total trajectory length L (the sum of all values dt for t = 1, …, N-1) [22]. If the direct distance between the trajectory’s first and last point is replaced by the maximum distance dist_*max*_ between any two points of the trajectory we obtain the radial effectiveness (= dist_max_ / L*)*. The radial effectiveness is illustrated in Fig 1A and 1B.

### 2.7 Statistical analysis

At least three biological experiments were analyzed. The data are presented as mean +/- standard deviation. One-sample or two-sample t-test was used for pairwise comparisons (IBM SPSS Statistics). The significant differences are indicated by *p<0.05, **p<0.01 or ***p<0.001.

## 3. Results

### 3.1 Effects of MET knockdown and afatinib treatment on receptor tyrosine kinases and intracellular signaling

We analyzed protein lysates after MET knockdown and afatinib treatment by Western blot to assess the influence on MET and EGFR signaling. The afatinib response was compared between MET knockdown (MET KD), control (Ctr) and non-transfected (NT) cells. DMSO was used as solvent control.

#### 3.1.1 Effects of MET knockdown and afatinib treatment on MET expression and activation

**Fig 2:**
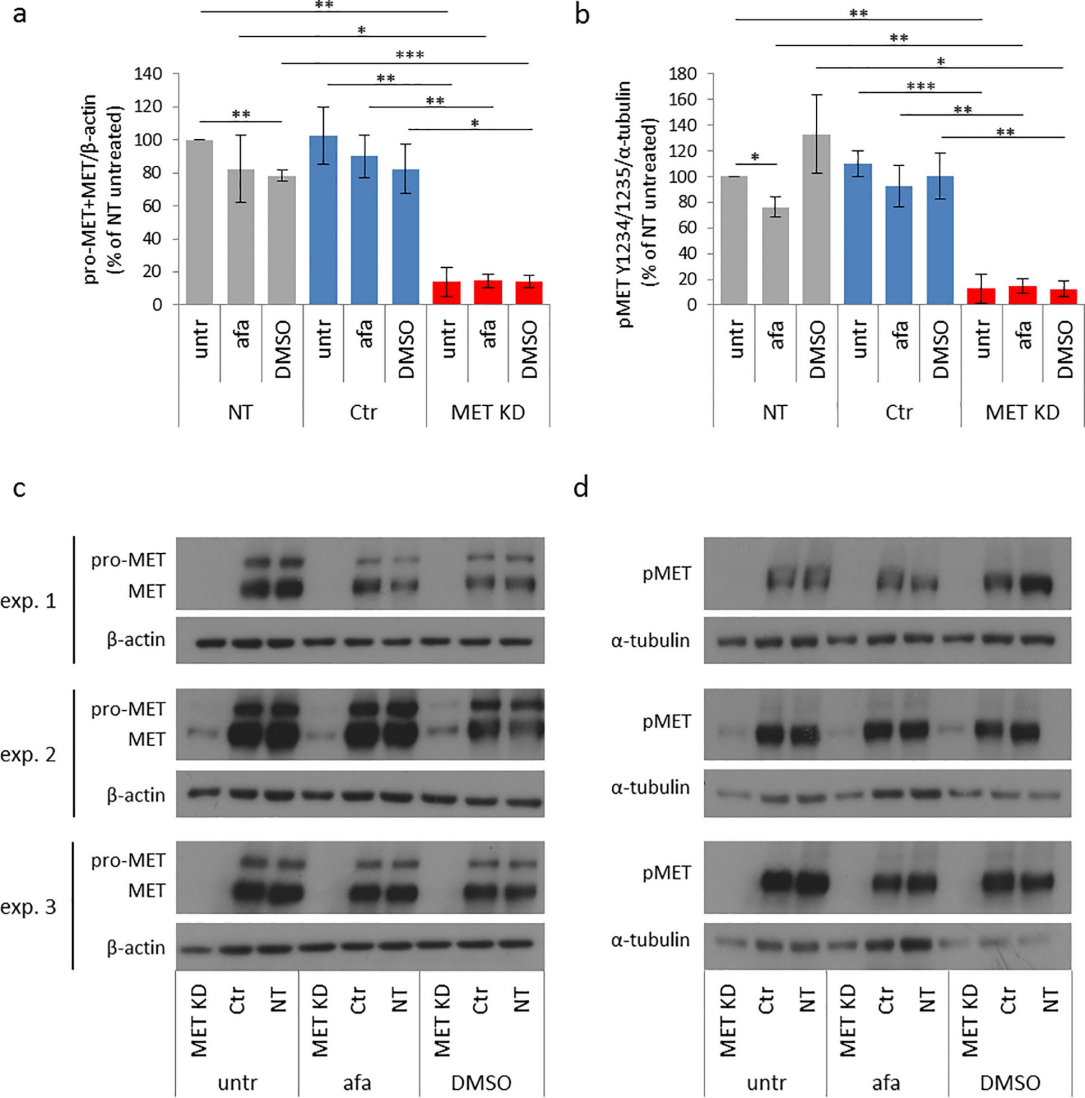
MET knockdown decreases MET expression and activation. Hs746T cells were transfected with MET siRNA (MET KD) or control siRNA (Ctr). Non-transfected cells (NT) were analyzed in parallel. Two days after transfection, cells were treated with 0.5 μM afatinib or 0.05% DMSO (solvent control) for 20 minutes. Cell lysates were analyzed by Western blot with antibodies against MET (a) and pMET Y1234/1235 (b). The MET antibody recognizes mature MET and its precursor pro-MET. The mean of three biological experiments with standard deviation is shown. T-test was used to compare afatinib-treated, DMSO-treated and untreated cells within each group (NT, Ctr, MET KD) and to compare the untreated, afatinib treated and DMSO treated cells between the groups. Statistically significant differences are indicated by *p<0.05, **p<0.01 or ***p<0.001. The corresponding Western blots for MET (c) and pMET Y1234/1235 (d) are shown.

After MET KD, MET expression was reduced to 14% compared to NT cells. Afatinib treatment did not influence MET expression in any of the three groups (NT, Ctr, MET KD). DMSO treatment reduced MET expression to 78% in non-transfected cells, but not in Ctr cells or MET KD cells (Fig 2A and 2C). The activation of MET in MET KD cells was reduced to 13% compared to NT cells. Afatinib treatment reduced MET activation to 76% in NT cells, but not in Ctr or MET KD cells. DMSO showed no effect on MET activation (Fig 2B and 2D).

#### 3.1.2 Effects of MET knockdown and afatinib treatment on EGFR expression and activation

**Fig 3:**
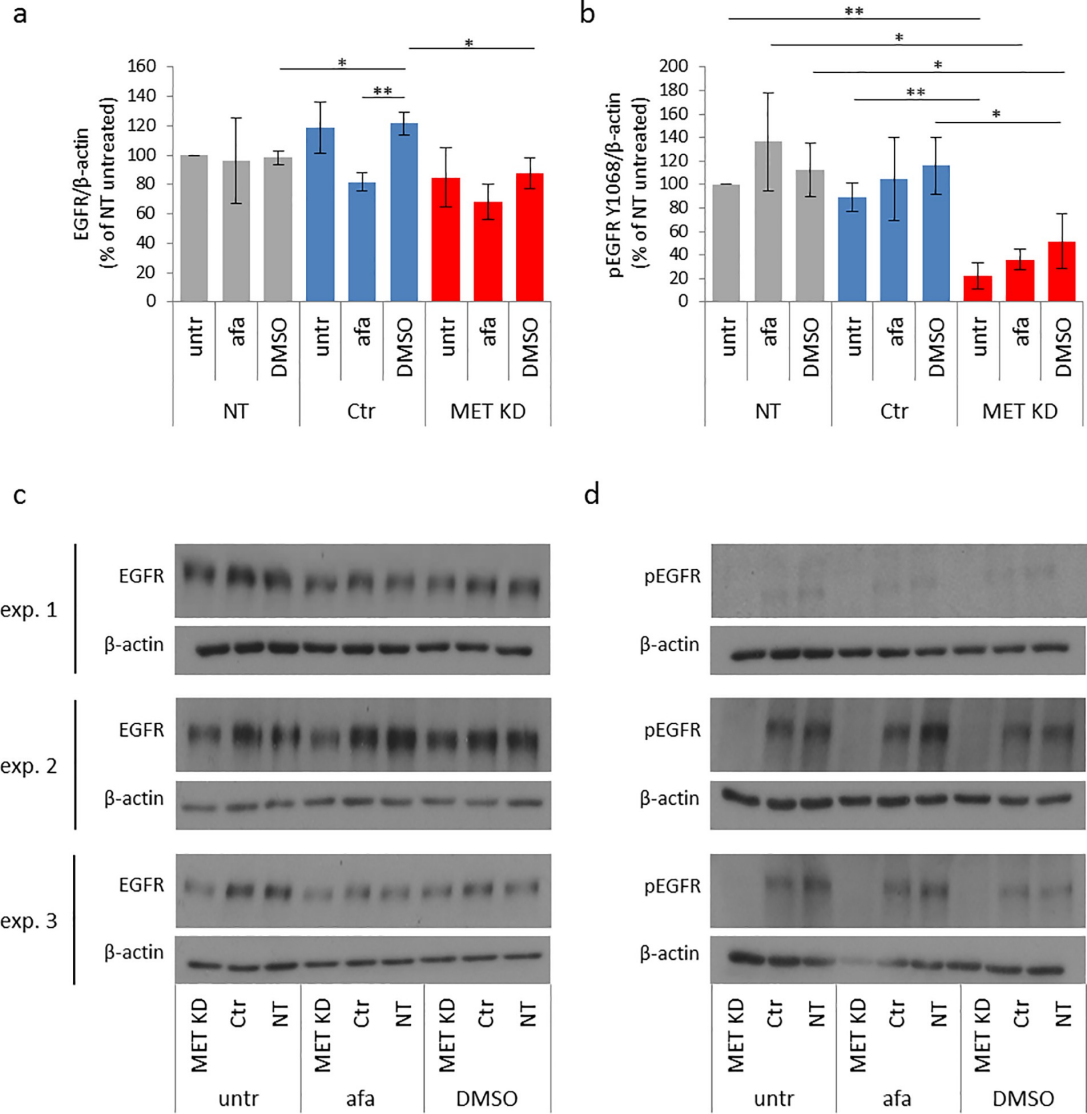
MET knockdown decreases EGFR activation and does not influence EGFR expression. Hs746T cells were transfected with MET siRNA (MET KD) or control siRNA (Ctr). Non-transfected cells (NT) were analyzed in parallel. Two days after transfection, cells were treated with 0.5 μM afatinib or 0.05% DMSO (solvent control) for 20 minutes. Cell lysates were analyzed by Western blot with antibodies against EGFR (a) and pEGFR Y1068 (b). The mean of three biological experiments with standard deviation is shown. T-test was used to compare afatinib-treated, DMSO-treated and untreated cells within each group (NT, Ctr, MET KD) and to compare the untreated, afatinib treated and DMSO treated cells between the groups. Statistically significant differences are indicated by *p<0.05 or **p<0.01. The corresponding Western blots for EGFR (c) and pEGFR Y1068 (d) are shown.

EGFR expression was not reduced after MET knockdown. Neither afatinib nor DMSO treatment showed any effect on EGFR expression (Fig 3A and 3C). MET knockdown resulted in reduction of EGFR activation to 22%, compared to NT cells. Neither afatinib nor DMSO had any influence on EGFR activation (Fig 3B and 3D).

#### 3.1.3 Effects of MET knockdown and afatinib treatment on MAPK and AKT activation

**Fig 4:**
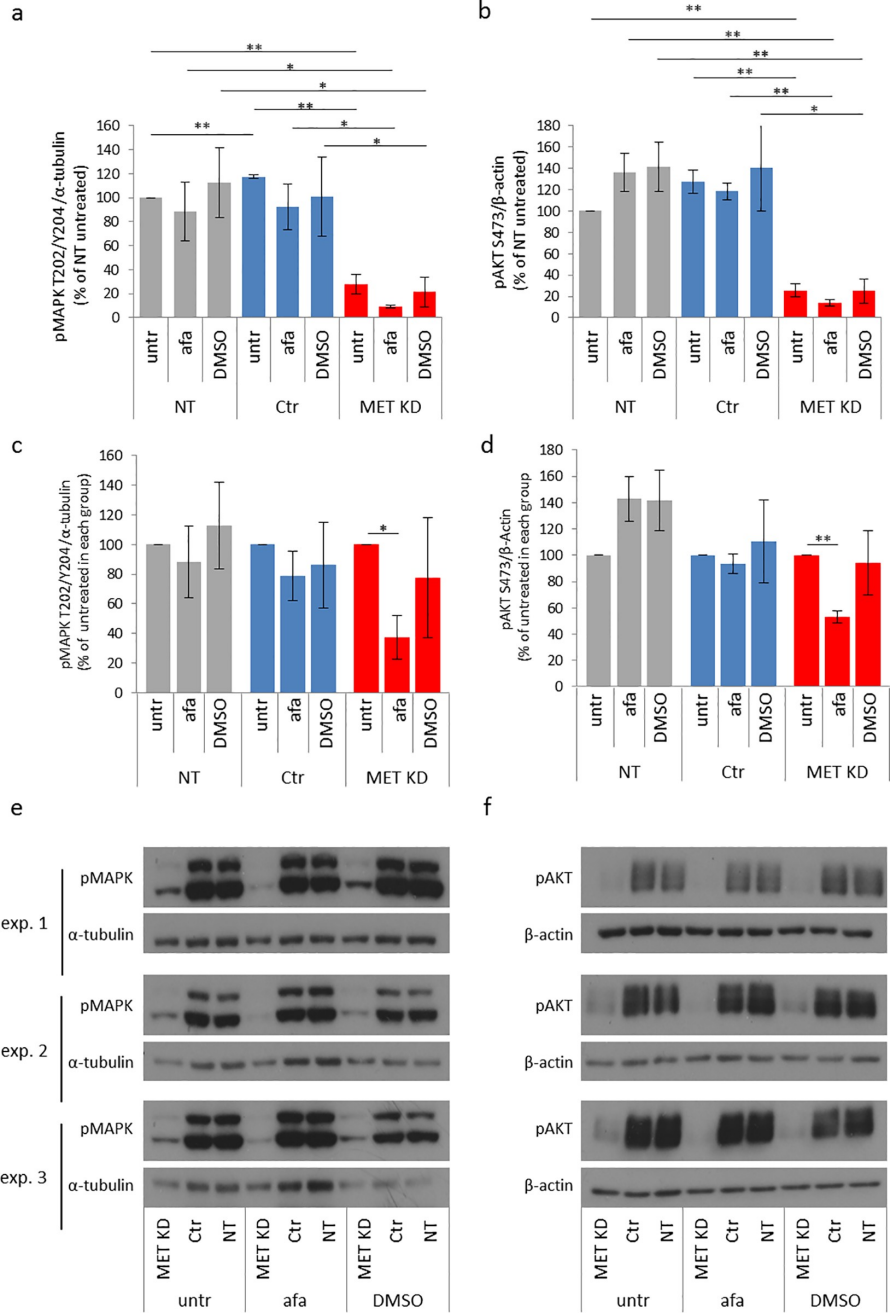
Afatinib decreases MAPK and AKT activation after MET knockdown. Hs746T cells were transfected with MET siRNA (MET KD) or control siRNA (Ctr). Non-transfected cells (NT) were analyzed in parallel. Two days after transfection, cells were treated with 0.5 μM afatinib or 0.05% DMSO (solvent control) for 20 minutes. Cell lysates were analyzed by Western blot with antibodies against pMAPK T202/Y204 (a,c) and pAKT S473 (b, d). Data are shown as percent of NT untreated cells (a, b) or as percent of untreated cells of each group (NT, Ctr, MET KD) (c, d). The mean of three biological experiments with standard deviation is shown. T-test was used to compare afatinib-treated, DMSO-treated and untreated cells within each group (NT, Ctr, MET KD) (a, b, c, d) and to compare the untreated, afatinib treated and DMSO treated cells between the groups (a, b). Statistically significant differences are indicated by *p<0.05 or **p<0.01. The corresponding Western blots for pMAPK T202/Y204 (e) and pAKT S473 (f) are shown.

After MET knockdown, activation of downstream kinases MAPK and AKT was reduced to 28% and 26% respectively, compared to NT cells (Fig 4A, 4B, 4E and 4F). To illustrate the effect of afatinib more clearly, data were depicted as a percentage relative to the untreated cells of each group (NT, Ctr, MET KD). Only in MET knockdown cells, afatinib treatment resulted in reduction of MAPK and AKT activation to 37% and 53% (Fig 4C and 4D).

Afatinib reduced activation of MAPK and AKT, but not EGFR in MET knockdown cells. One would expect that reduced EGFR activation results in reduced MAPK and AKT activation. However, small, not detectable changes in EGFR activation could be enough to result in changed MAPK and AKT activation. Within these experiments, only the pEGFR Y1068 phosphorylation site was analyzed. In earlier experiments using the proteome profiler, we compared effects of afatinib on EGFR phosphorylation using site-specific and pan EGFR antibodies. EGFR Y1086 phosphorylation was not reduced after afatinib treatment in NCI-N87 cells, but phosphorylations of AKT and MAPK were reduced. The treatment effect of afatinib was more pronounced with the use of a pan EGFR antibody, which detects multiple EGFR phosphorylation sites [5].

Taken together, the results indicate that knockdown of MET in Hs746T cells reduced the activation of MET and EGFR as well as the activation of downstream kinases MAPK and AKT. Moreover, only the MET KD cells responded to afatinib treatment as shown by decreased MAPK and AKT activation.

### 3.2 Confirmation of MET knockdown during cell proliferation assay and time-lapse microscopy

In order to assess the success of MET knockdown during phenotypic assays, protein lysates were generated in parallel. The expression of MET was analyzed by Western blot.

**Fig 5:**
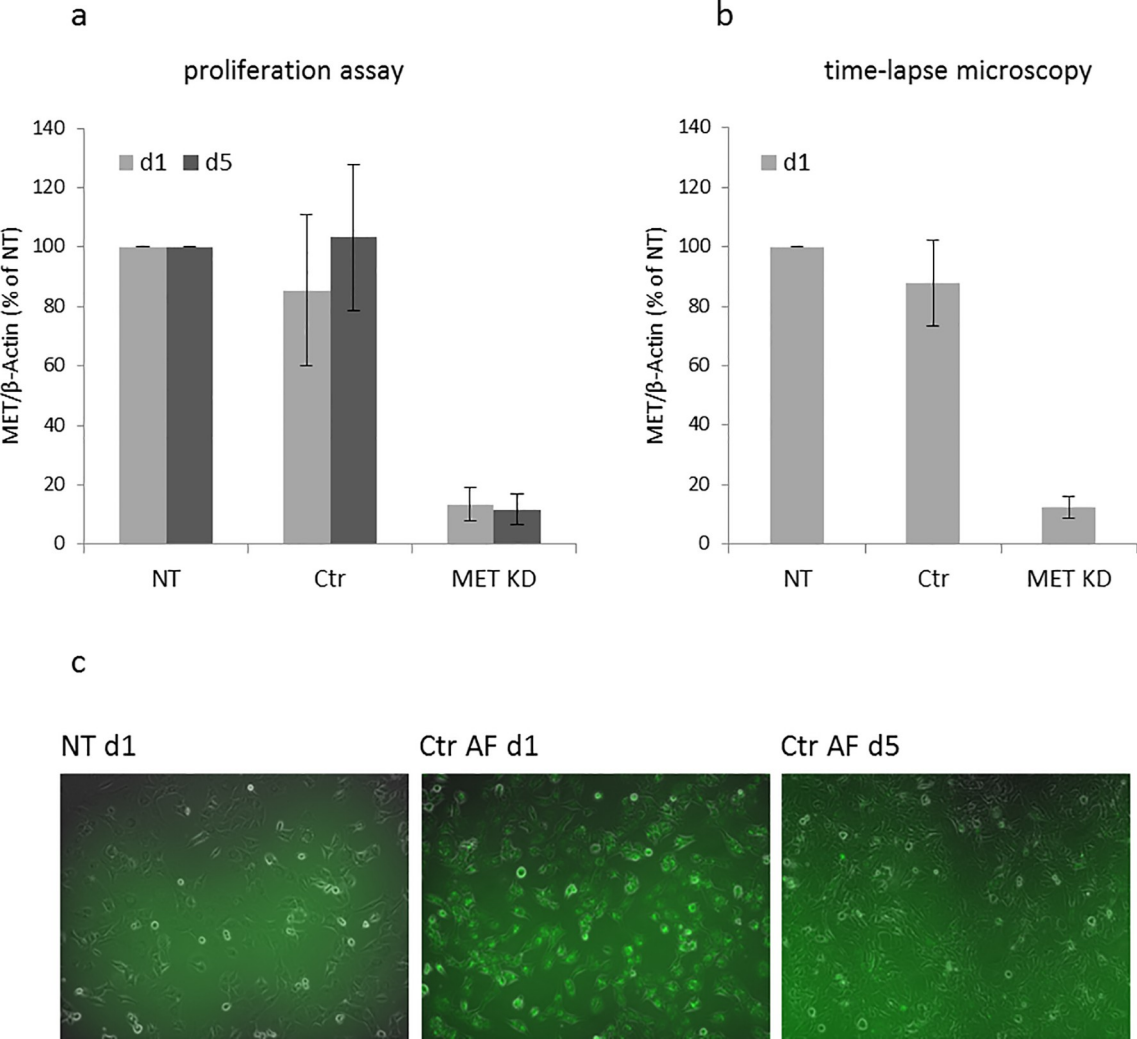
Confirmation of MET knockdown during phenotypic assays. Hs746T cells were transfected with MET siRNA (MET KD) or control siRNA (Ctr). Non-transfected cells (NT) were analyzed in parallel. MET knockdown was confirmed for the duration of cell proliferation assay (a) and time-lapse microscopy (b) by Western blot. MET knockdown on day 1 (d1) after transfection corresponds to plating of cells for proliferation assay and knockdown on day 5 (d5) corresponds to the end of the assay. For time-lapse microscopy, MET knockdown corresponds to the assay start. The mean of three biological experiments with standard deviation is shown. The presence of Alexa Fluor labeled siRNA control (Ctr AF) was monitored on d1 and d5 (c).

During cell proliferation assay, MET expression was reduced to 13% compared to NT cells on day 1 and 12% on day 5 (Fig 5A). At the start of time-lapse microscopy, MET expression was reduced to 12% compared to NT cells (Fig 5B). Transfection efficiency of at least 90% on day 1 after transfection was observed by fluorescence microscopy. On day 5 after transfection, the labeled siRNA was no longer visible (Fig 5C). However, the effect of siRNA on MET knockdown was still detectable on day 5 (Fig 5A).

Thus, we demonstrated successful MET knockdown during the duration of proliferation and motility assays.

### 3.3 Effects of MET knockdown and afatinib treatment on cell proliferation

In order to investigate the effects of MET knockdown and afatinib treatment on proliferation, we performed the WST-1 cell proliferation assay. The afatinib response on proliferation (measured as metabolic activity) was compared between MET KD, Ctr and NT cells. DMSO was used as solvent control.

**Fig 6:**
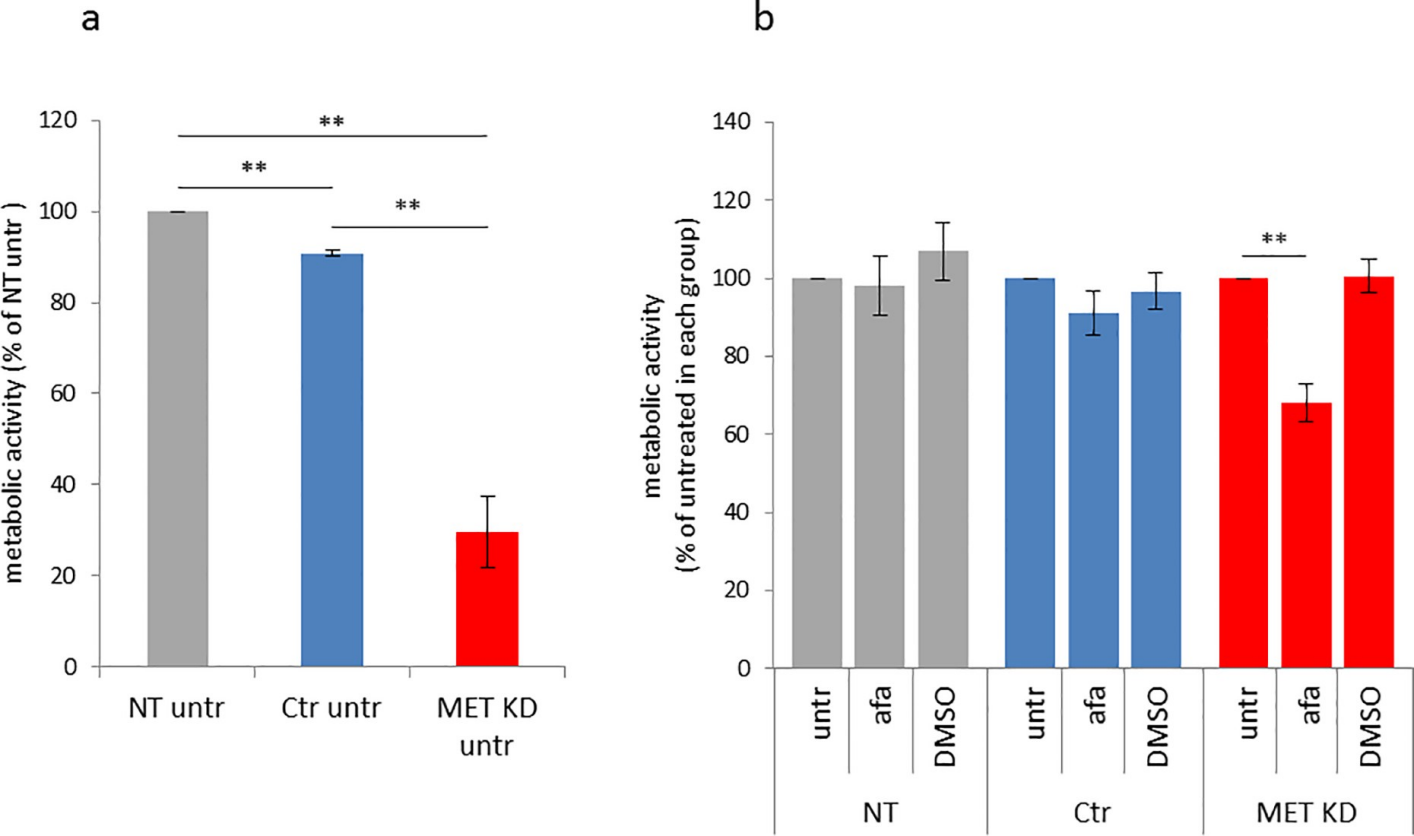
Afatinib reduces cell proliferation after MET knockdown. Hs746T cells were transfected with MET siRNA (MET KD) or control siRNA (Ctr). Non-transfected cells (NT) were analyzed in parallel. Cells were harvested and re-plated one day after transfection. The following day, cells were treated with 0.5 μM afatinib or 0.05% DMSO for 72 hours. The mean of three biological experiments with standard deviation is shown. Data are shown as percent of NT untreated cells (a) or as percent of untreated cells of each group (NT, Ctr, MET KD) (b). T-test was used to compare the untreated cells between the groups and to compare afatinib-treated, DMSO-treated and untreated cells within each group (NT, Ctr, MET KD). Statistically significant differences are indicated by **p<0.01.

The metabolic activity of MET knockdown cells was reduced to 30% in comparison to NT cells (Fig 6A). Afatinib reduced the metabolic activity to 69% in MET KD cells, but not in NT or Ctr cells. DMSO showed no effect on metabolic activity (Fig 6B). As comparison, the afatinib-responsive gastric cancer cell lines MKN1 and NCI-N87 showed under similar conditions a reduction in proliferation to 65% and 35%, respectively [5].

We showed reduced proliferation after MET KD in Hs746T cells. Furthermore, only the MET KD cells were sensitive to afatinib treatment.

### 3.4 Effects of MET knockdown on motility parameters

The effects of MET knockdown on cellular motility were assessed by time-lapse microscopy. MET KD, Ctr and NT cells were filmed for 7 hours. Different motility parameters were assessed by manual and computer-driven analysis.

#### 3.4.1 Assessment of motility parameters

**Fig 7:**
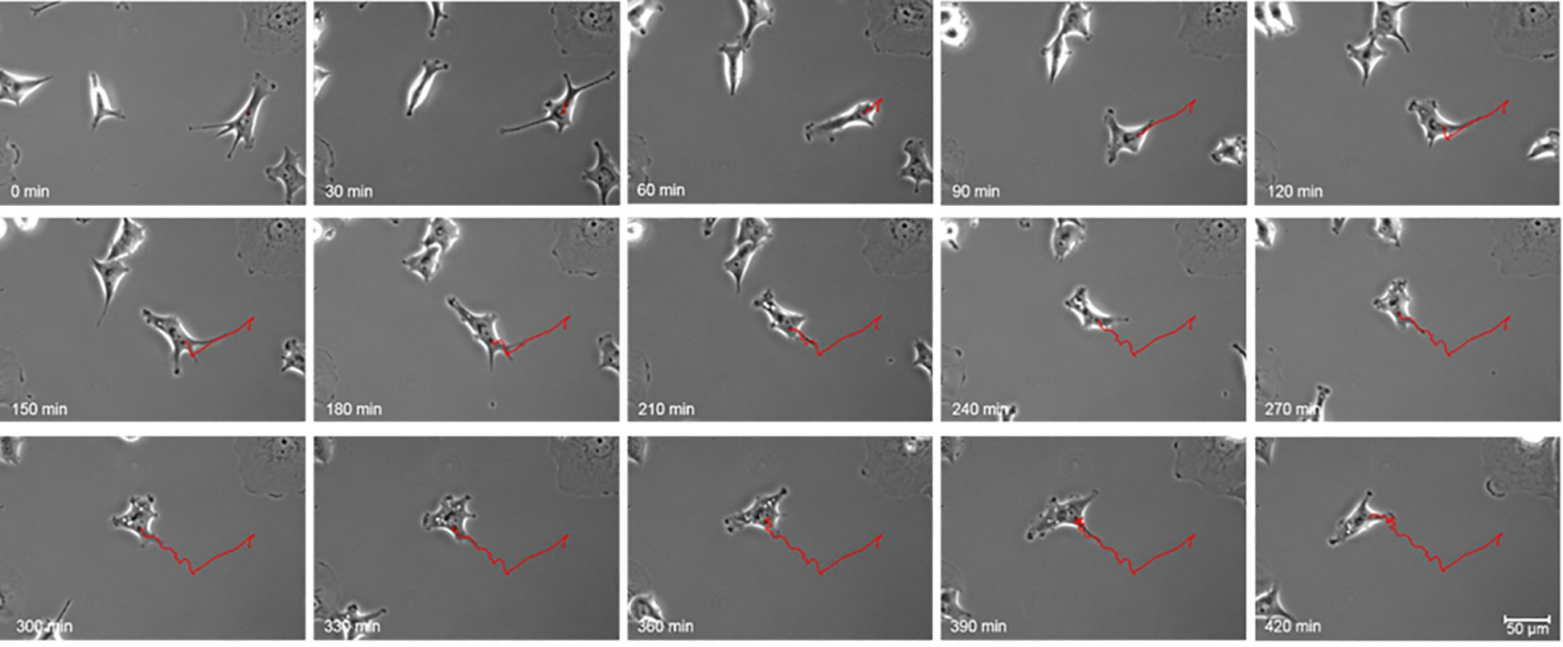
Illustration of cell movement. Hs746T cell movement was tracked for 7 hours. The position of one exemplary cell in 30 minute intervals is depicted. The red line indicates the trajectory of the cell.

The manual analysis of motility parameters allowed the assessment of motile cell number and approximate average speed. A cell was defined as motile when it completely left the field previously occupied by the cell itself. The movement of each cell was tracked while the film was running and the approximate average speed was calculated. To determine the approximate average speed manually the cell was not tracked at each point in time but its path is marked on the screen while the video is running (Fig 7). In addition, a computational, software-driven tracking and analysis were performed (cf. Section 2.6). Thus, xyt data was generated meaning the position on the x- and y-axis of each cell is captured for each of the 141 time points (t). This allows the assessment of further motility parameters and a more precise calculation of the average speed. The calculated parameters are explained in Fig 1.

#### 3.4.2 Effects of MET knockdown on cell motility, speed, displacement entropy and radial effectiveness

**Fig 8:**
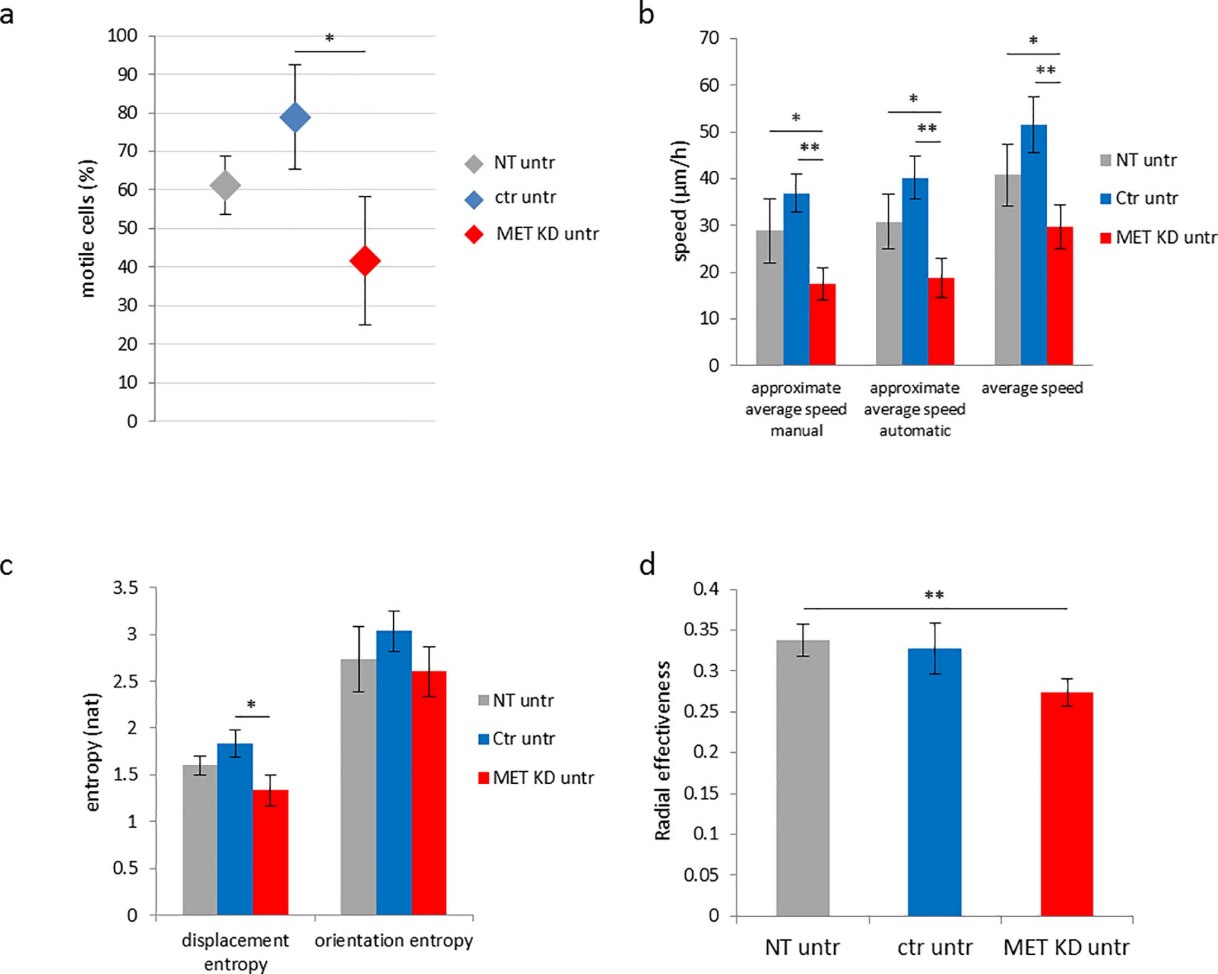
MET knockdown decreases cell motility, speed, displacement entropy and radial effectiveness. Hs746T cells were transfected with MET siRNA (MET KD) or control siRNA (Ctr). Non-transfected cells (NT) were analyzed as control. Time-lapse microscopy was started one day after transfection. Cell movement was tracked for 7 hours to assess motility (a), average speed (b), entropy (c) and radial effectiveness (d). The mean of three to four biological experiments with standard deviation is shown. T-test was used to compare NT, MET KD and Ctr cells. Statistically significant differences are indicated by *p<0.05 or **p<0.01.

**Fig 9:**
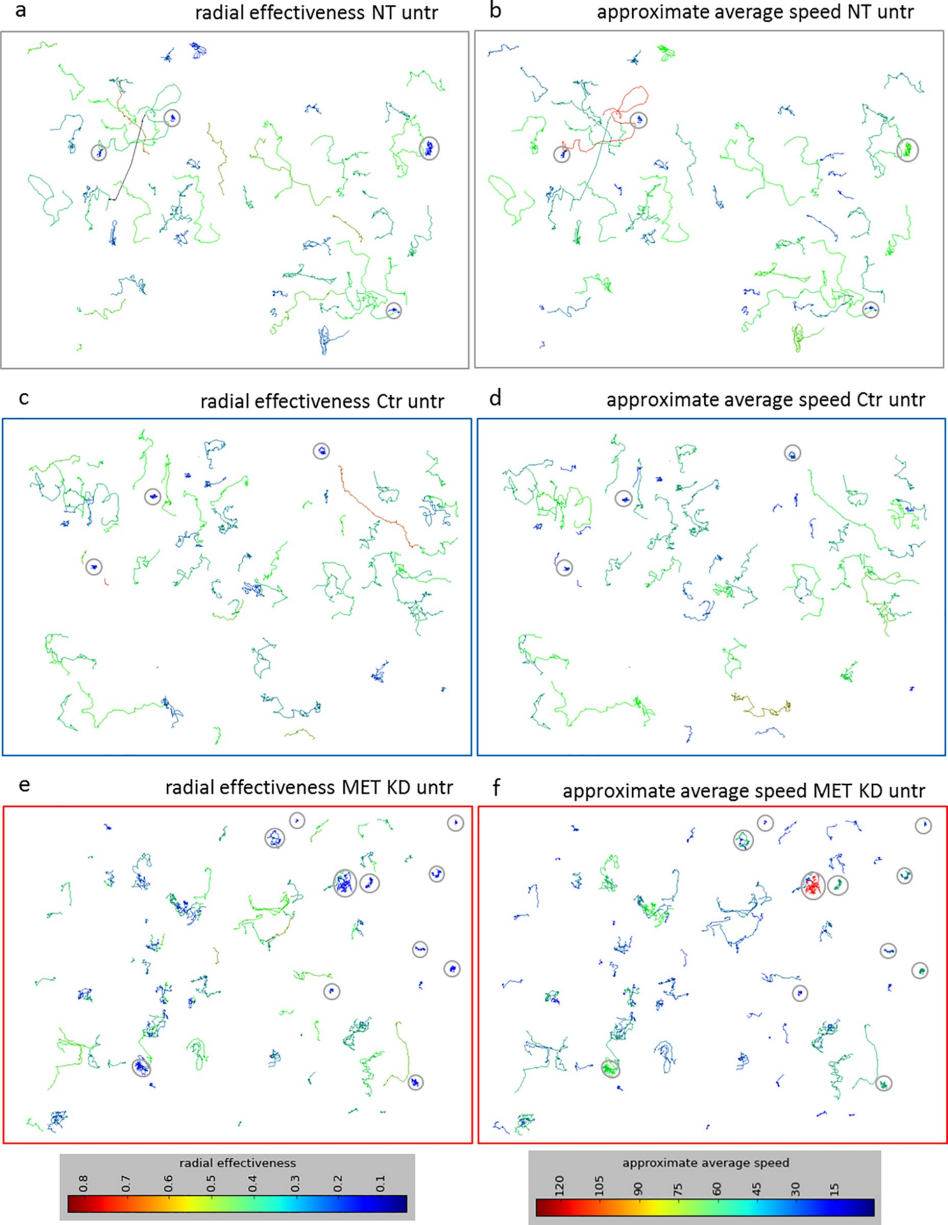
MET knockdown increases proportion of cells with low radial effectiveness but medium approximate average speed. Hs746T cells were transfected with MET siRNA (MET KD) or control siRNA (Ctr). Non-transfected cells (NT) were analyzed as control. Time-lapse microscopy was started one day after transfection. Cell movement was tracked for 7 hours to assess radial effectiveness and approximate average speed. The trajectories of one exemplary film for each condition are shown. The trajectories were color-coded for radial effectiveness (panels a, c, d) or approximate average speed (panels b, d, f). The cells with lowest radial effectiveness (<0.1) were highlighted with grey circles.

**Fig 10:**
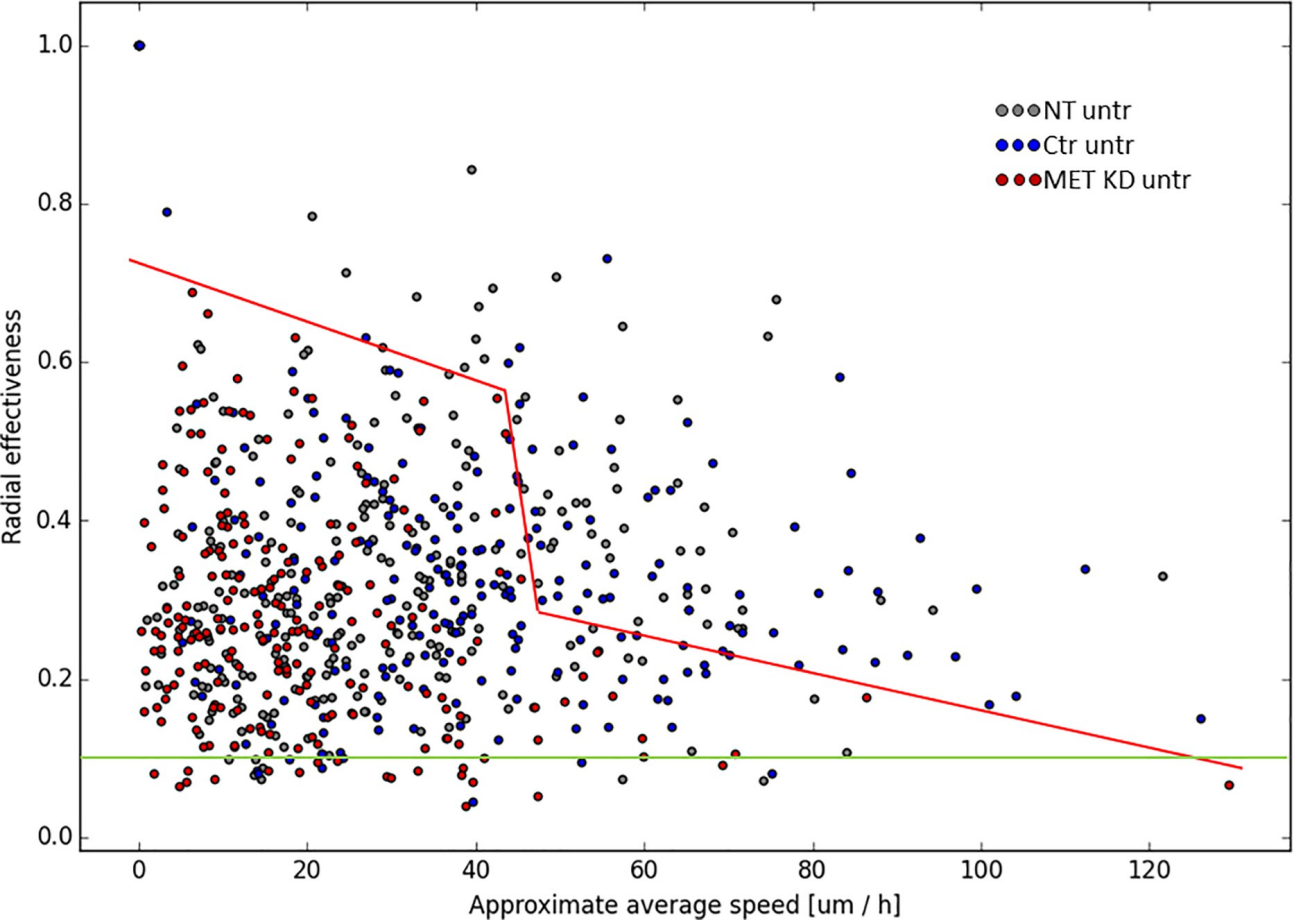
Relation between radial effectiveness and approximate average speed. Hs746T cells were transfected with MET siRNA (MET KD) or control siRNA (Ctr). Non-transfected cells (NT) were analyzed as control. Time-lapse microscopy was started one day after transfection. Cell movement was tracked for 7 hours. The radial effectiveness of each cell was plotted against approximate average speed. The MET KD cells are depicted in red, the Ctr ones in blue and the NT cells in grey. The red line delimits the red circles towards the right hand side illustrating, for instance, that for a radial effectiveness above 0.24 only Ctr and NT cells are assuming an approximate average speed >46 μm/h. The green straight line is defined such that 5% of all cells have a radial effectiveness below the value corresponding to that line, namely below 0.100.

After MET knockdown 42% of cells were classified as motile, whereas 79% of cells were classified as motile after control transfection. The percentage of motile cells in non-transfected cells was 61%. The motility reduction in MET knockdown cells compared to control cells was significant, while the decrease was not statistically significant when compared to non-transfected cells (Fig 8A). The approximate average speed (manual) in MET KD cells (18 μm/h) was reduced compared to Ctr cells (37 μm/h) and NT cells (29 μm/h). The automatic assessment of approximate average speed resulted in similar levels. The average speed (automatic) was reduced in MET KD cells (30 μm/h) compared to Ctr cells (52 μm/h) and NT cells (41 μm/h) (Fig 8B). The displacement entropy in MET KD cells was reduced compared to Ctr cells. The orientation entropy after MET KD was similar compared to NT and Ctr cells (Fig 8C). Knockdown of MET resulted in reduced radial effectiveness compared to NT cells and Ctr cells. However, only the difference between MET KD cells and NT cells was significant (Fig 8D). Fig 9 illustrates the trajectories of 3 selected films. The colors indicate different levels of radial effectiveness or approximate average speed, respectively. After MET KD, the trajectories are shorter which results in reduced approximate average speed. Moreover, there is a high proportion of tangle-like trajectories. This observation can be described by calculation of radial effectiveness. 5% of the in total investigated cells have a radial effectiveness value of lower than 0.10. In MET KD cells, 11/105 (10.5%) cells showed small values (<0.10) for radial effectiveness whereas 3/94 (3.2%) in Ctr cells and 4/59 (6.8%) in NT cells showed small values (grey circles) (Fig 9A, 9C and 9E, one example per condition). In Ctr and NT cells, cells with low radial effectiveness showed predominantly low approximate average speed (indicated as blue trajectory). Whereas in MET KD cells, such cells showed to a considerable extend medium/high approximate average speed values (indicated as green/red trajectory) (Fig 9B, 9D and 9F). The relationship between radial effectiveness and approximate average speed for all films is illustrated in Fig 10. The MET KD cells cluster together in the area of low radial effectiveness and low approximate average speed. Some MET KD cells showed high approximate average speed values, but only in combination with relatively low radial effectiveness (cf. red line in Fig 10). After MET KD 20/213 (9.4%) of cells showed low radial effectiveness (<0.10), whereas 7/252 (2.8%) of NT cells and 8/218 (3.7%) of Ctr cells showed low radial effectiveness.

To sum up, the percentage of motile cells as well as the motility parameters (approximate) average speed, displacement entropy and radial effectiveness were reduced after MET KD in Hs746T cells. A high proportion of MET KD cells demonstrated an altered, tangle-like movement behavior.

## 4. Discussion

### 4.1 MET as resistance factor for afatinib and other anti-HER therapies

To investigate the importance of the receptor tyrosine kinase MET as resistance factor we used the gastric cancer cell line Hs746T, which bears a *MET* amplification and *MET* exon 14 skipping due to a splice-site mutation. The binding site of CBL (Y1003), which mediates MET degradation, is missing due to exon 14 skipping. Consequently, MET oncogenic signaling is sustained [23, 24]. We previously demonstrated that Hs746T cells are resistant to afatinib treatment [5]. In the present work, we show that afatinib resistance in Hs746T cells can be reversed by MET knockdown. Consequently, MET is identified as a resistance factor for afatinib therapy.

MET is currently under scrutiny for its role as resistance factor for anti-HER therapies in different entities. Experiments in lung cancer cell lines (H358, H1650, H1975) revealed an effect of MET inhibition with su11274 and afatinib treatment on cell growth and apoptosis. Treatment of lung cancer cells with a combination of MET siRNA and EGFR siRNA enhanced the growth inhibiting effect of EGFR siRNA alone [25].

*In vivo* generated afatinib-resistant clones of H1975 lung cancer cells showed decreased expression of EGFR, HER2, HER3 and HER4 and increased expression of MET, c-KIT and PDGFRβ. The combined but not the single or double knockdown of HER3, c-KIT and MET resulted in cell death [26]. This indicates that MET also plays an important role in acquired afatinib resistance.

In a 3D culture system of colorectal cancer cells, the phosphorylation of MET and RON was higher in cells with weaker response to EGFR-directed antibody cetuximab. Furthermore, this resistance was reversed by treatment with MET/RON TKI crizotinib [27].

The two *HER2*-amplified gastric cancer cell lines NCI-N87 and SNU-216 were sensitive to EGFR tyrosine kinase inhibitor lapatinib. In presence of MET ligand HGF, these cells were resistant to lapatinib. MET knockdown reversed the lapatinib resistance after HGF stimulation. Moreover, the resistance was also reversed by treatment with a MET inhibitor. The authors conclude that MET is an important resistance factor in these cell lines [28]. Although the cell lines used in this study show no amplification of *MET*, MET knockdown improved sensitivity to lapatinib.

Data from our group in combination with GDSC (Genomics of Drug Sensitivity in Cancer) dataset show a correlation of afatinib sensitivity and MET Y1234/1235 activation. Gastric cancer cell lines with higher IC50 value for afatinib sensitivity showed stronger MET Y1234/1235 activation. Moreover, the three *MET*-amplified cell lines MKN45, Hs746T and SNU-5 were amongst the 4 cell lines with the highest IC50 value [5, 29].

Afatinib as well as the combination of trastuzumab and afatinib were tested in a cohort of 32 patients with disease progression on trastuzumab. *MET* amplification was not found in any of the patients. However, the analysis of post-afatinib progression sites of one patient revealed a *MET* amplification which was not present in the analyzed tumor before treatment. The response of one post-afatinib progression site to a combination treatment of afatinib and AMG 337 (MET inhibitor) was demonstrated in a xenograft model [30].

Our observation that afatinib resistance in a *MET*-amplified gastric cancer cell line can be reversed by MET knockdown is in line with data from studies using different entities or anti-HER therapeutics. Taken together, MET is a resistance factor for anti-HER therapies in different cancer entities, including gastric cancer. Studies with cell lines without *MET* amplification indicate that it is the high MET expression and not necessarily the amplification that may lead to resistance to anti-HER therapies. One might argue that using one cell line is not sufficient to show the relevance of MET as resistance factor. However, as indicated in this paragraph, the relevance of MET as resistance factor to EGFR-targeted therapies was already shown in various cell lines, including gastric cancer cell lines, and in clinical samples.

### 4.2 MET as therapeutic target in gastric cancer

MET knockdown resulted in reduced signal transduction, cell proliferation and motility. We conclude that Hs746T cells are addicted to MET, according to the concept of oncogene addiction [31]. Although the concept is well known, to our knowledge we were the first to show decreased intracellular signaling, proliferation and motility after MET knockdown in Hs746T cells.

MET knockdown in two *MET*-amplified lung cancer cell lines (EBC-1, H1993) resulted in growth inhibition. This growth inhibiting effect was not observed in cell lines without *MET* amplification, with lower MET expression and no basal MET activation [32]. In H358, H1650 and H1975 lung cancer cell lines, the inhibition of MET showed only weak effects on cell growth and apoptosis. When MET siRNA was used, only weak effects on cell growth were observed [25]. The lack of *MET* amplification in these cell lines could explain the minor response to MET inhibition.

MET inhibitors were also tested in gastric cancer cell lines. The selective MET inhibitor KRC-00715 suppressed tumor growth in a Hs746T mouse xenograft model [33]. Anchorage-independent growth of three *MET*-amplified gastric cancer cell lines (MKN45, SNU-5, KATOIII) was reduced after treatment with MET inhibitors PHA-665752 or crizotinib [34]. The analysis of 49 gastric cancer cell lines revealed that *MET* amplification predicts the sensitivity to MET inhibitors. Six *MET*-amplified gastric cancer cell lines, amongst them Hs746T, were sensitive to both MET antibodies and MET TKIs in cell viability assays [35]. This study confirmed the earlier results from Smolen *et al*. who showed sensitivity of *MET*-amplified gastric cancer cells lines to the MET inhibitor PHA-665752. They further showed decreased viability after MET knockdown in *MET*-amplified cell line SNU-5 [36]. Effects of MET inhibition in advanced gastric cancer were further observed in patient-derived xenograft (PDX) models. MET TKI volitinib showed antitumor activity in cases with high MET expression and activation [8].

To date, no drug targeting the HGF/MET pathway has been approved for gastric cancer treatment, but antibodies or tyrosine kinase inhibitors against MET are currently being investigated in clinical trials (see introduction). Both MET antibody ABT-700 and tyrosine kinase inhibitor AMG-337, seem to be more effective in tumors with *MET* amplification [14, 15]. Besides *MET* amplification, also *MET* exon 14 skipping is being discussed as a possible marker for anti-MET therapies. In lung cancer, patients with *MET* exon 14 skipping responded to MET inhibitors [37, 38]. In gastric cancer, this correlation has not yet been examined.

Different *in vitro*, xenograft and clinical studies came to the same conclusion that *MET*-amplification is an indicator for oncogene addiction to MET and consequently for the efficacy of MET inhibitors. Our own MET knockdown data in *MET*-amplified cells support these observations.

### 4.3 The importance of MET for cell motility and speed

It has been shown that targeting tumor cell motility may be a strategy against invasion and metastasis [39]. In the present study, the percentage of motile cells, the approximate average speed, the average speed, the displacement entropy and the radial effectiveness were reduced after MET knockdown in Hs746T cells. Moreover, we observed a high proportion of cells with low radial effectiveness after MET knockdown, especially in case of simultaneously medium/high approximate average speed, demonstrating the altered movement behavior. This altered movement is visible as a tangle pattern of the trajectories. The transfection itself also influenced the motility parameters. The number of motile cells, approximate average speed, average speed, displacement entropy and orientation entropy were slightly higher in Ctr cells compared to NT cells.

Activation of MET signaling cascade by its ligand HGF results in colony dispersal, epithelial-mesenchymal transition (EMT) and increased motility [40]. The complex biological program triggered by MET activation is also called “invasive growth” and includes the promotion of cell proliferation and cell invasion, as well as apoptosis protection [41]. Amongst others, the ERK/MAPK and PI3K/AKT pathways are important downstream mediators of MET activation [40].

Our data confirm the important role of MET as well as MAPK and PI3K/AKT pathways, as regulators of cell motility and consequently of invasion and metastasis.

### 4.4 Combination of afatinib and anti-MET therapies

In our experiments, cell proliferation and downstream signaling were at the lowest following combined MET knockdown and afatinib treatment. Thus, the combined inhibition of EGFR, HER2, HER3 and MET is most effective with regard to inhibition of proliferation.

The expression of MET, EGFR and HER2 in 293 patients with unresectable or recurrent gastric or gastroesophageal junction cancer was examined by immunohistochemistry. The combined overexpression of MET, HER2 and EGFR was observed in 3% of gastric tumors. 5% of tumors were MET- and HER2-positive, but EGFR-negative. The co-overexpression of MET and EGFR without HER2 overexpression was present in 12% of tumors. In total, 20% of gastric cancers were either MET-, EGFR- and HER2- positive or MET- and EGFR- or HER2-positive [42]. For these patients, a combination of pan-HER inhibitor afatinib and MET-directed antibody or inhibitor could be beneficial. As mentioned before, the combination treatment of afatinib and MET inhibitor AMG 337 resulted in complete tumor response in a xenograft model with one post-afatinib, *MET* amplified progression site from one gastric cancer patient [30].

In malignant pleural mesothelioma, the combination of afatinib and MET/ALK/RON/ROS inhibitor crizotinib has been investigated in cell culture and mouse xenograft models. In both systems, the combination was more effective than each drug alone in suppressing cell proliferation [43]. The combination of afatinib and crizotinib showed promising results also in an acquired resistance setting. The viability of lung cancer cells with acquired afatinib resistance was markedly reduced after afatinib and crizotinib treatment. Moreover, tumor growth was inhibited by a combination of afatinib and crizotinib in a mouse xenograft model with acquired afatinib resistance [44].

### 4.5 Conclusion

Based on *in vitro* studies, pan HER tyrosine kinase inhibitor afatinib is a promising therapy option for advanced gastric cancer. However, the presence of resistance factors should be considered before therapy. In the present work, we identified *MET* amplification as a resistance factor for afatinib therapy in gastric cancer cell line Hs746T. Moreover, we identified MET as an important regulator of cell motility. Future clinical trials should investigate the importance of MET as a resistance factor for afatinib therapy and other HER-targeting therapies.

## Supporting information

S1 Raw Images (PDF)

## Abbreviations

afa: Afatinib
CBL: Casitas B-lineage Lymphoma
Ctr: Control
DMSO: Dimethyl sulfoxide
EGFR: Epidermal growth factor receptor
EMT: Epithelial-mesenchymal transition
GEJ: Gastroesophageal junction
MET: Hepatocyte growth factor receptor
HER: Human epidermal growth factor receptor
KD: Knockdown
MAPK: Mitogen-activated protein kinase
NT: Non-transfected
PDX: Patient-derived xenograft
PRAS40: Proline-rich Akt substrate of 40 kDa
AKT: Protein kinase B
untr: Untreated
