# Supplementary material for: MET as resistance factor for afatinib therapy and motility driver in gastric cancer cells
Source: PLoS One. 2019 Sep 26;14(9):e0223225. doi: 10.1371/journal.pone.0223225 (PMC6763200; doi:10.1371/journal.pone.0223225)
Supplement: S1 Raw Images — (PDF) [file pone.0223225.s001.pdf]

Figure 1a,c

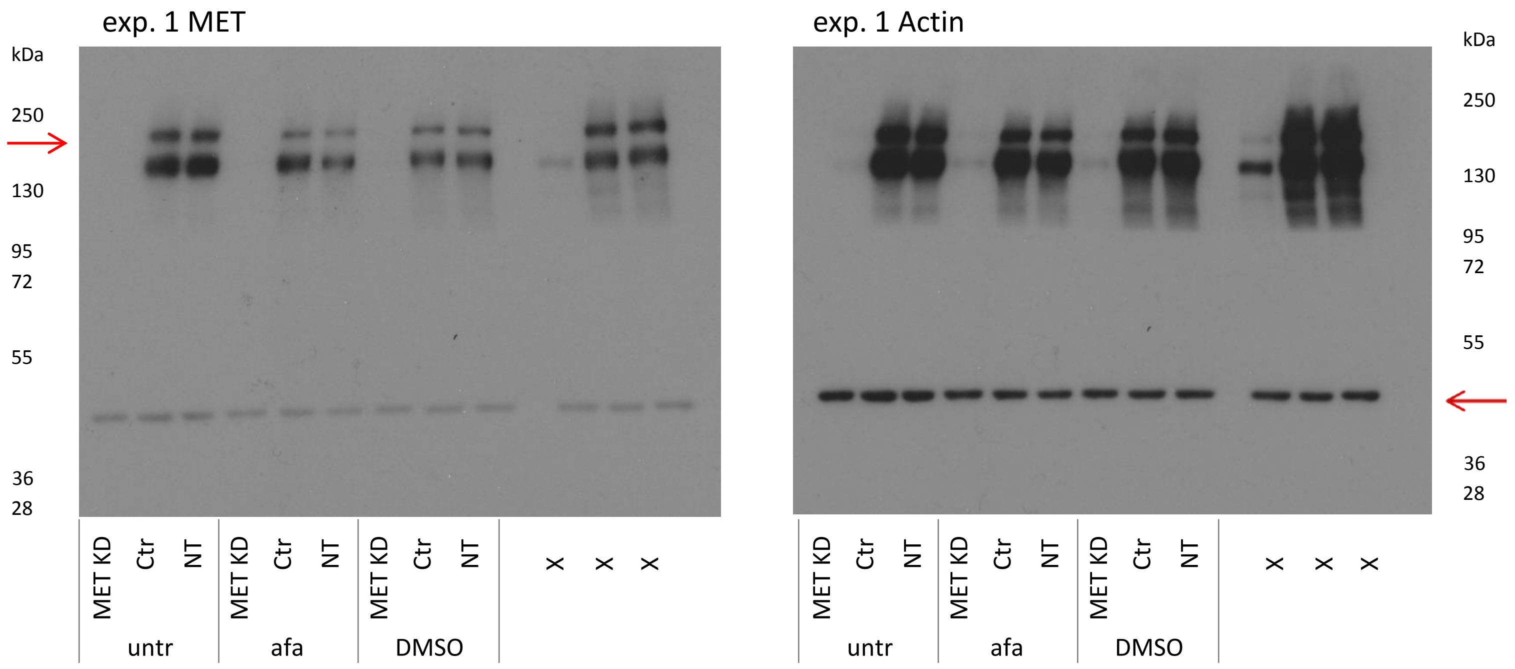

- All membranes were cut between 95 and 72 kDa
- The upper parts were stained with MET, pMET, EGFR or pEGFR antibodies
- The lower parts were stained with pMAPK and Tubulin or pAKT and Actin antibodies
- Different exposure times were analyzed for different proteins. For each protein, the images of the evaluated exposure times are depicted.

Figure 1a,c

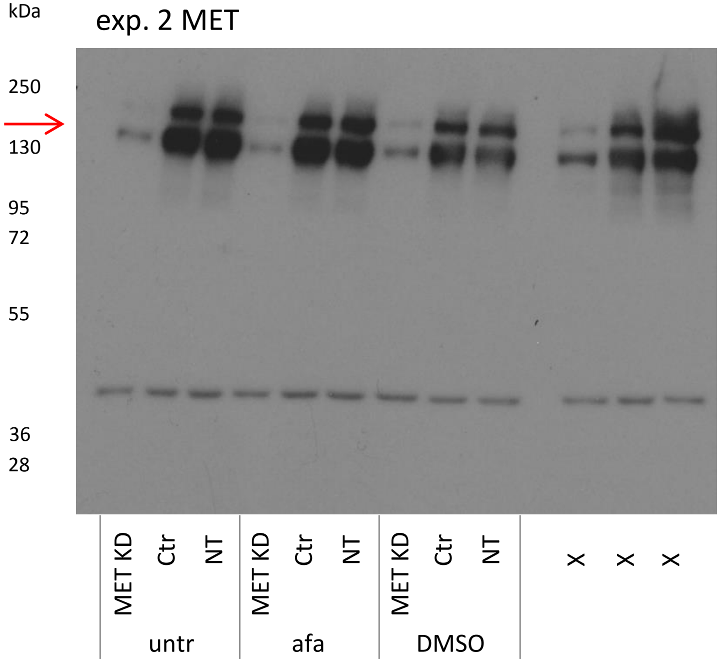

kDa
exp. 2 MET
250
130
95
72
55
36
28
MET KD
Ctr
NT
MET KD
Ctr
NT
MET KD
Ctr
NT
X
X
X
untr
afa
DMSO

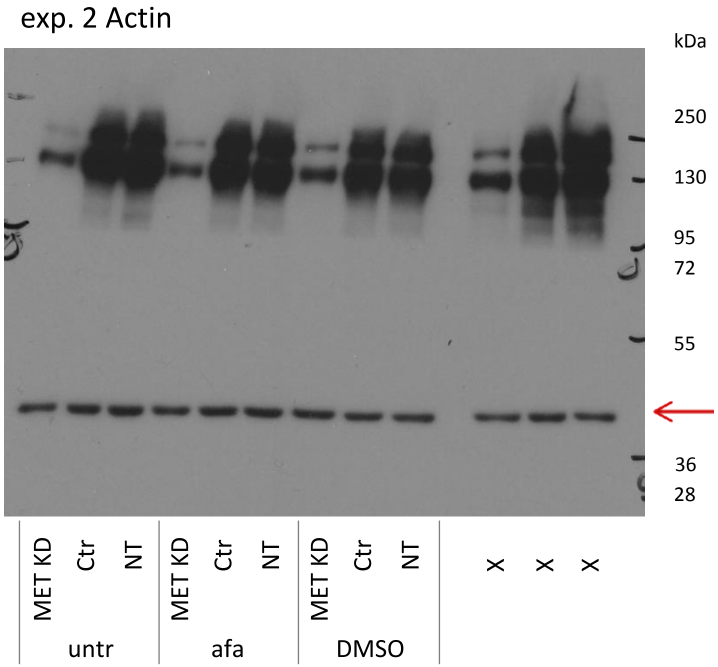

exp. 2 Actin
kDa
250
130
95
72
55
36
28
MET KD
Ctr
NT
MET KD
Ctr
NT
MET KD
Ctr
NT
X
X
X
untr
afa
DMSO

Figure 1a,c

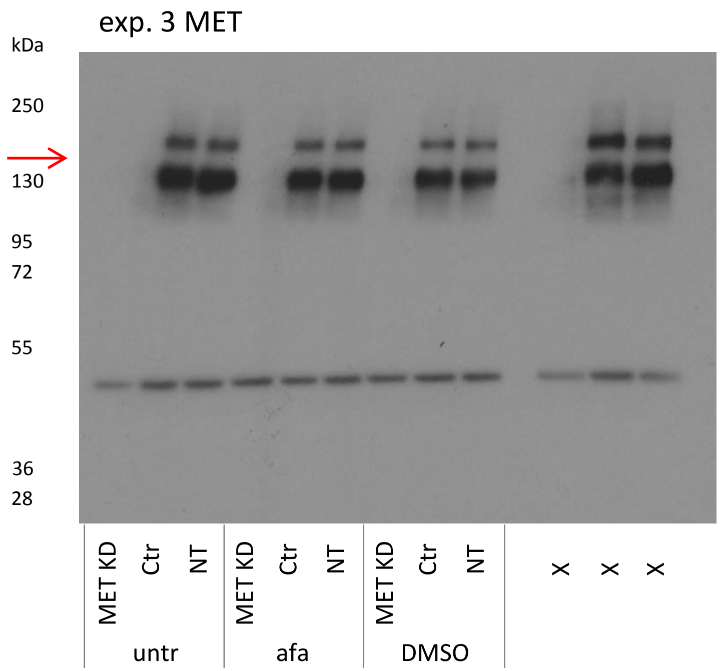
exp. 3 MET
kDa
250
130
95
72
55
36
28
MET KD
Ctr
NT
MET KD
Ctr
NT
MET KD
Ctr
NT
X
X
X
untr
afa
DMSO

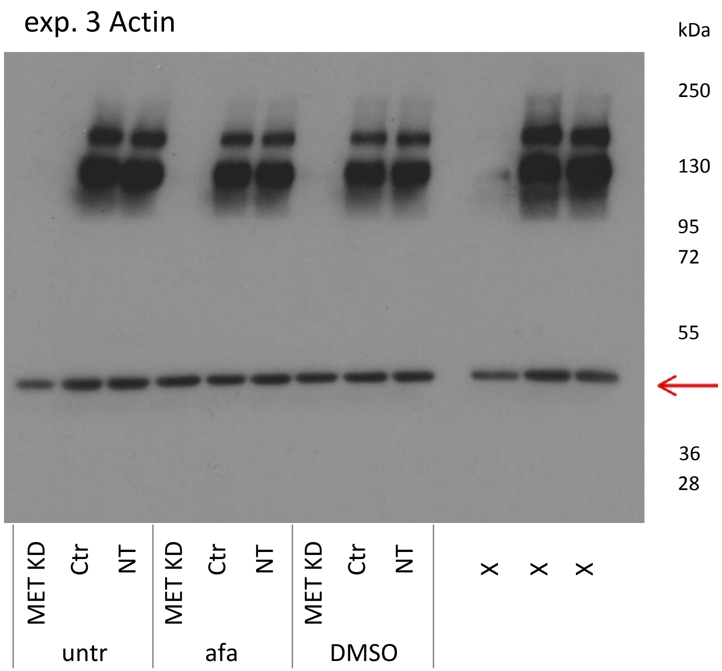
exp. 3 Actin
kDa
250
130
95
72
55
36
28
MET KD
Ctr
NT
MET KD
Ctr
NT
MET KD
Ctr
NT
X
X
X
untr
afa
DMSO

Figure 1b,d

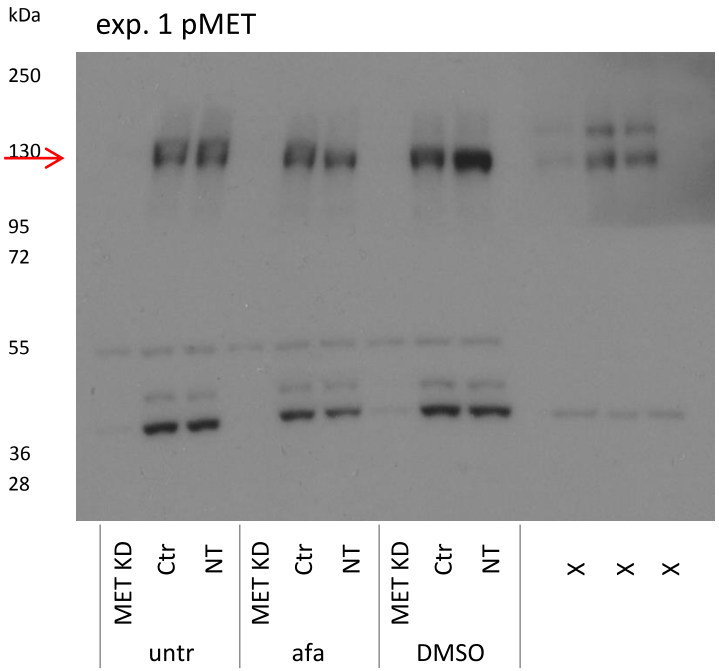
kDa
exp. 1 pMET
250
130
95
72
55
36
28
MET KD
Ctr
NT
MET KD
Ctr
NT
MET KD
Ctr
NT
X
X
X
untr
afa
DMSO

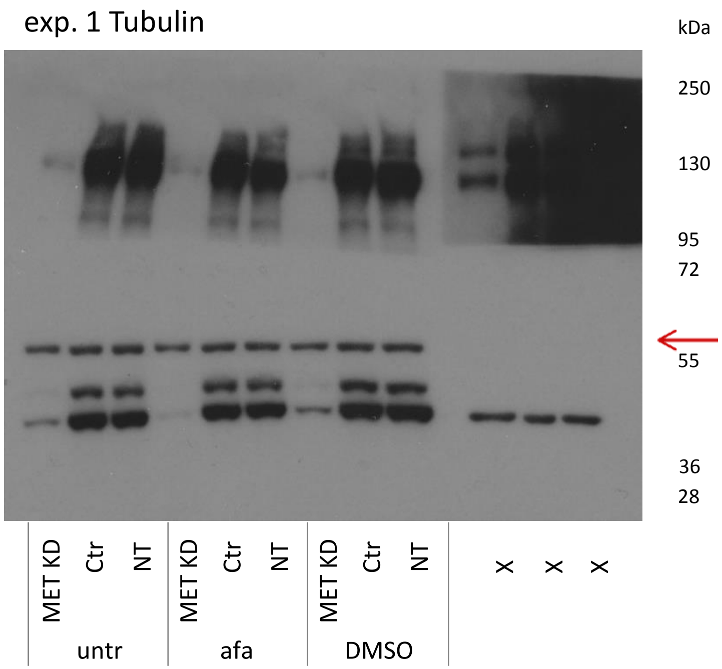
exp. 1 Tubulin
kDa
250
130
95
72
55
36
28
MET KD
Ctr
NT
MET KD
Ctr
NT
MET KD
Ctr
NT
X
X
X
untr
afa
DMSO

Figure 1b,d

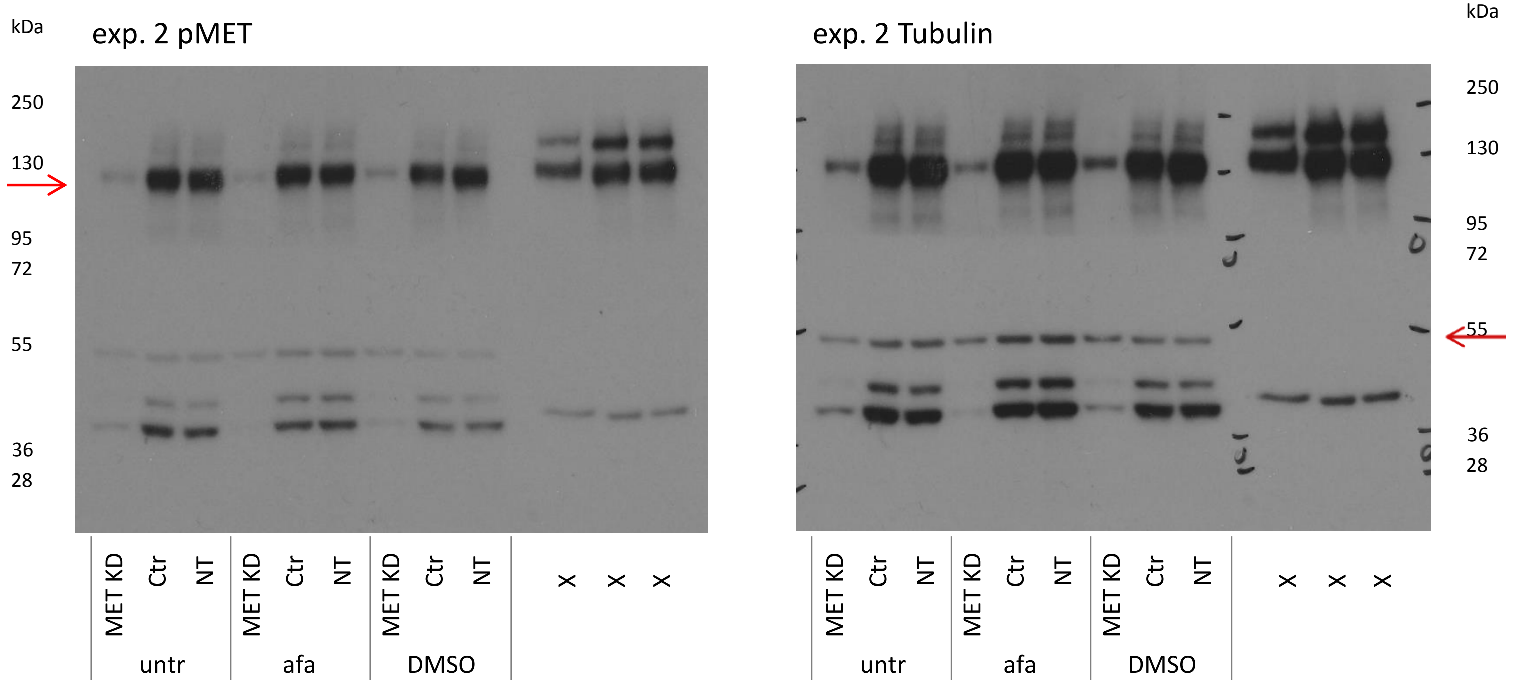
exp. 2 pMET
kDa
250
130
95
72
55
36
28
MET KD
Ctr
NT
MET KD
Ctr
NT
MET KD
Ctr
NT
X
X
X
untr
afa
DMSO
exp. 2 Tubulin
kDa
250
130
95
72
55
36
28
MET KD
Ctr
NT
MET KD
Ctr
NT
MET KD
Ctr
NT
X
X
X
untr
afa
DMSO

Figure 1b,d

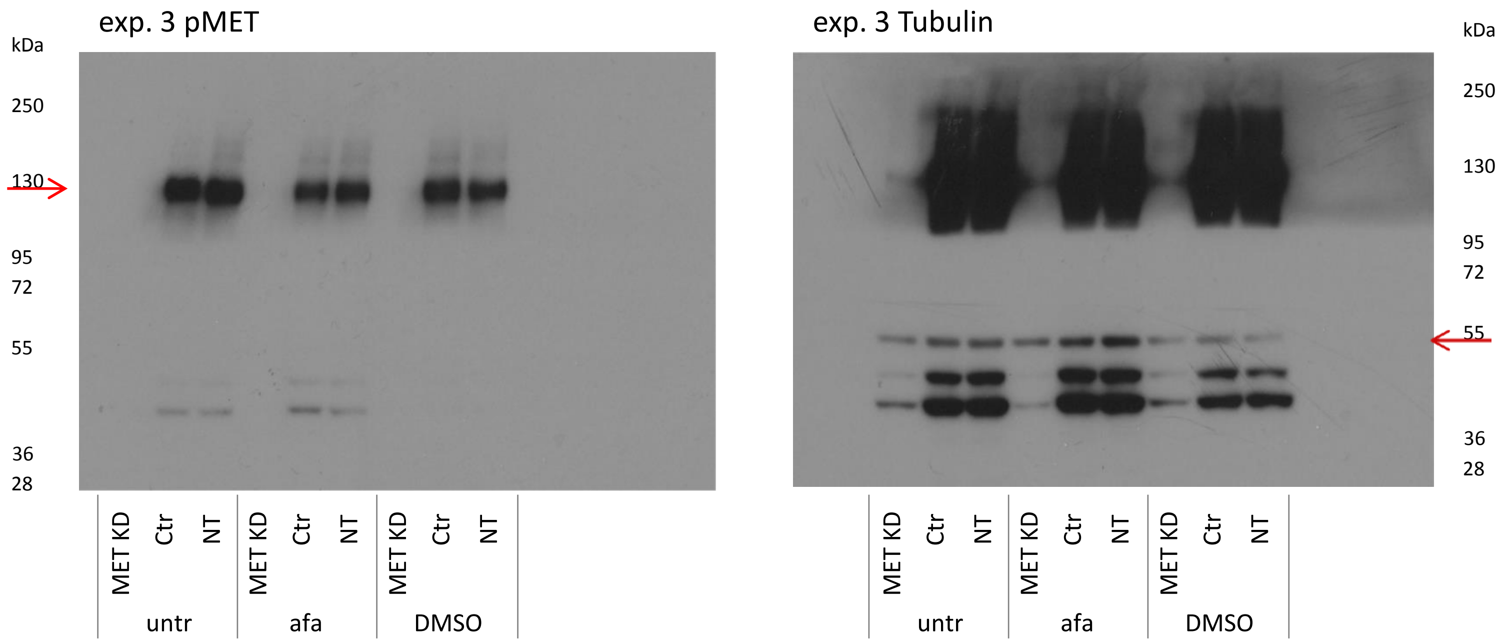
exp. 3 pMET
kDa
250
130
95
72
55
36
28
MET KD
Ctr
NT
MET KD
Ctr
NT
MET KD
Ctr
NT
untr
afa
DMSO
exp. 3 Tubulin
kDa
250
130
95
72
55
36
28
MET KD
Ctr
NT
MET KD
Ctr
NT
MET KD
Ctr
NT
untr
afa
DMSO

Figure 2a,c

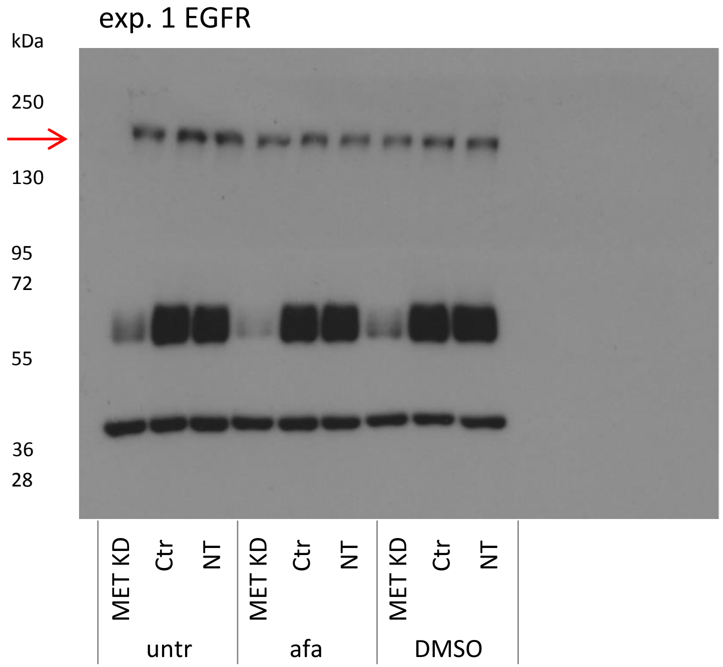
exp. 1 EGFR
kDa
250
130
95
72
55
36
28
MET KD
Ctr
NT
MET KD
Ctr
NT
MET KD
Ctr
NT
untr
afa
DMSO

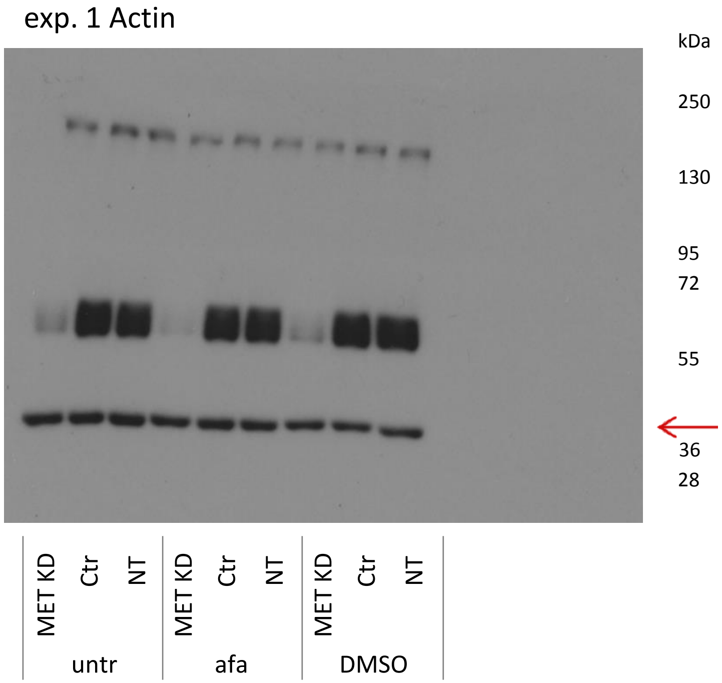
exp. 1 Actin
kDa
250
130
95
72
55
36
28
MET KD
Ctr
NT
MET KD
Ctr
NT
MET KD
Ctr
NT
untr
afa
DMSO

Figure 2a,c

exp. 2 EGFR

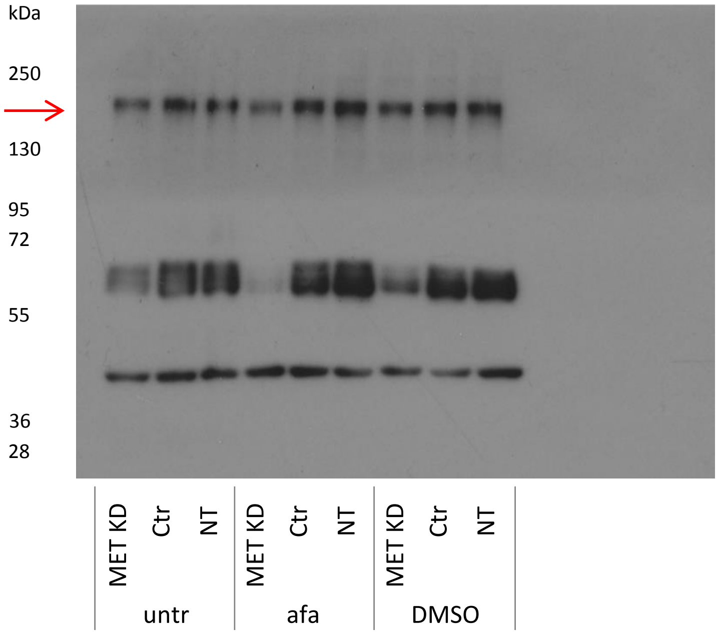

exp. 2 Actin

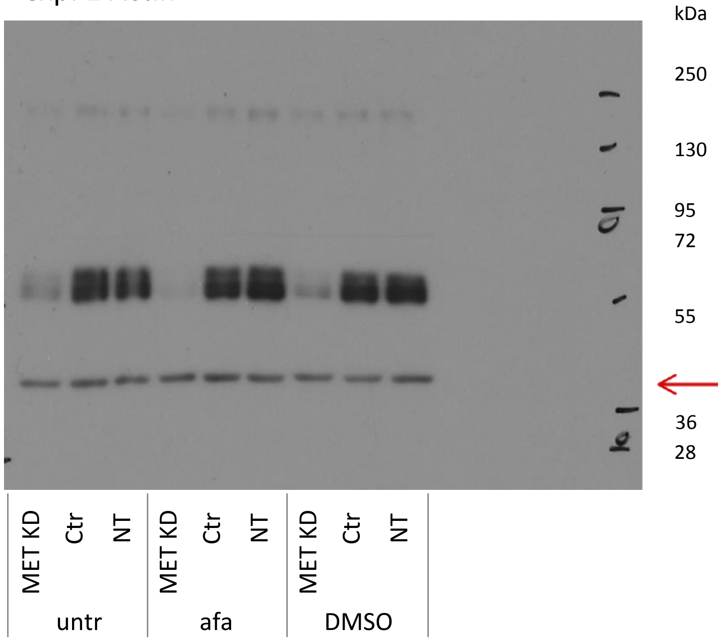

Figure 2a,c

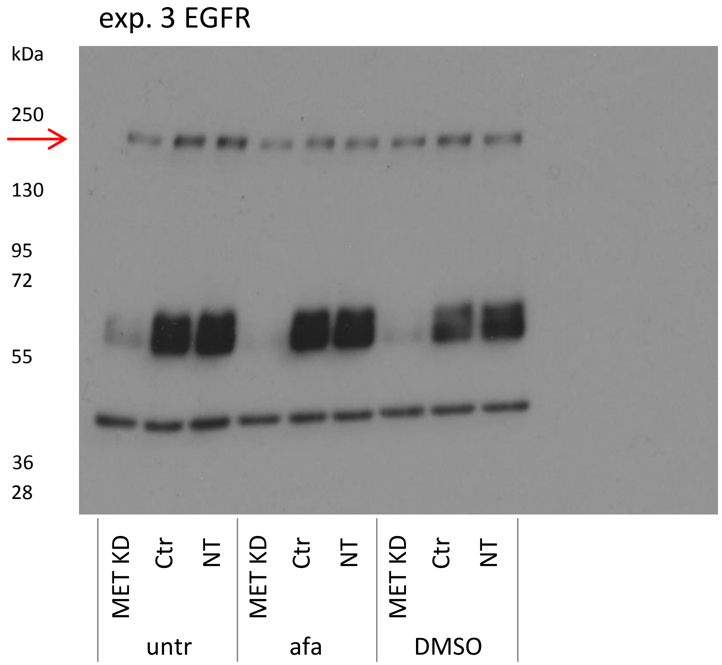
exp. 3 EGFR
kDa
250
130
95
72
55
36
28
MET KD
Ctr
NT
MET KD
Ctr
NT
MET KD
Ctr
NT
untr
afa
DMSO

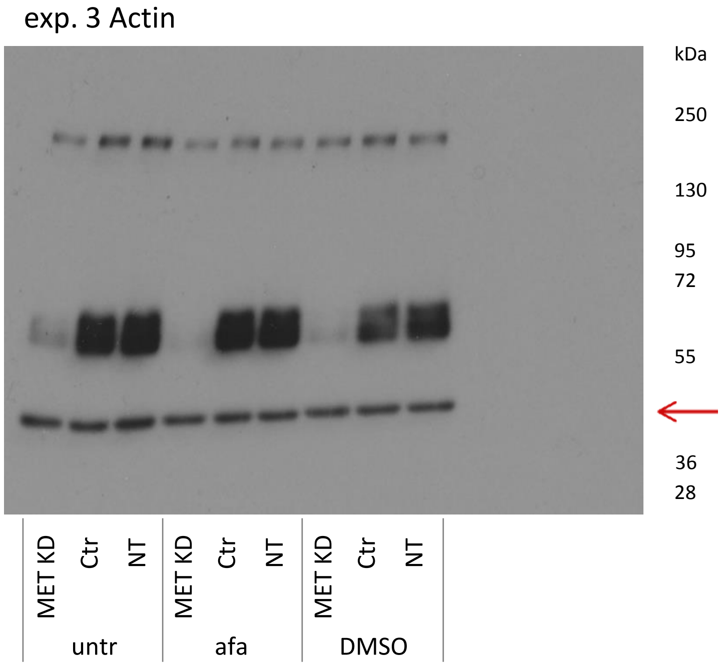
exp. 3 Actin
kDa
250
130
95
72
55
36
28
MET KD
Ctr
NT
MET KD
Ctr
NT
MET KD
Ctr
NT
untr
afa
DMSO

Figure 2b,d

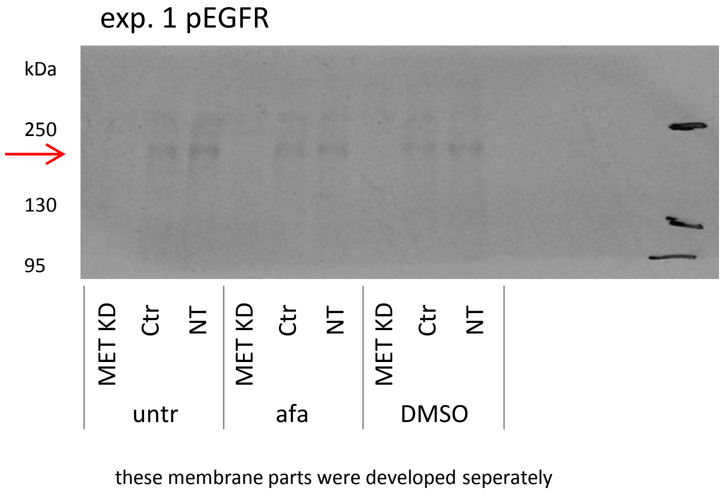
exp. 1 pEGFR
kDa
250
130
95
MET KD
Ctr
NT
MET KD
Ctr
NT
MET KD
Ctr
NT
untr
afa
DMSO
these membrane parts were developed seperately

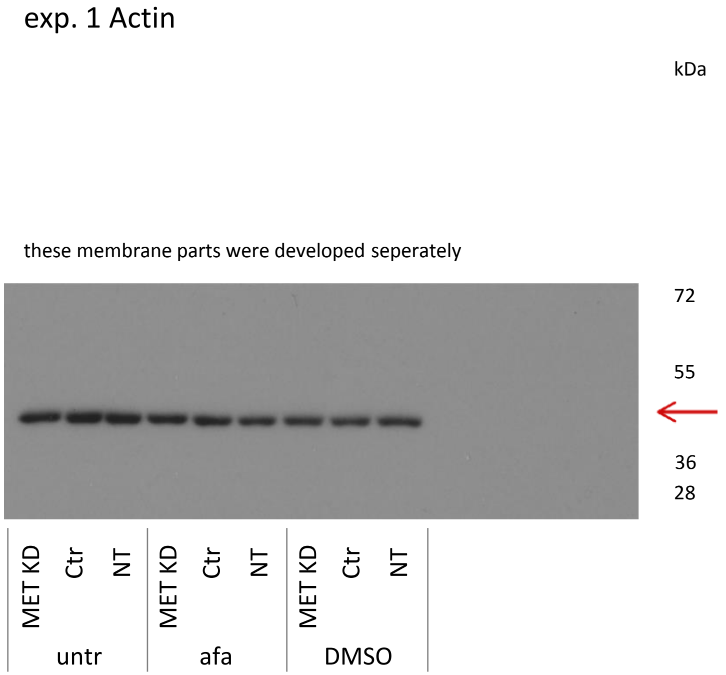
exp. 1 Actin
kDa
these membrane parts were developed seperately
72
55
36
28
MET KD
Ctr
NT
MET KD
Ctr
NT
MET KD
Ctr
NT
untr
afa
DMSO

Figure 2b,d

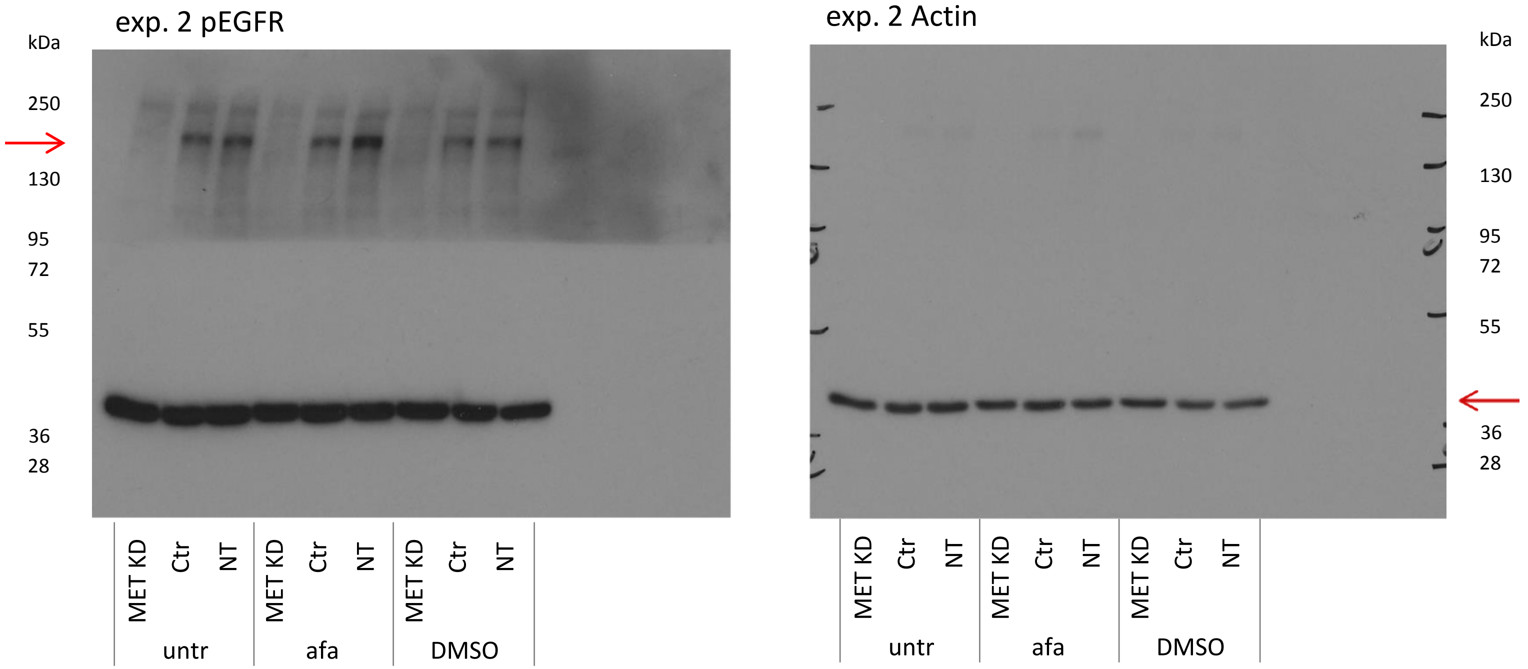
exp. 2 pEGFR
kDa
250
130
95
72
55
36
28
MET KD
Ctr
NT
MET KD
Ctr
NT
MET KD
Ctr
NT
untr
afa
DMSO
exp. 2 Actin
kDa
250
130
95
72
55
36
28
MET KD
Ctr
NT
MET KD
Ctr
NT
MET KD
Ctr
NT
untr
afa
DMSO

Figure 2b,d

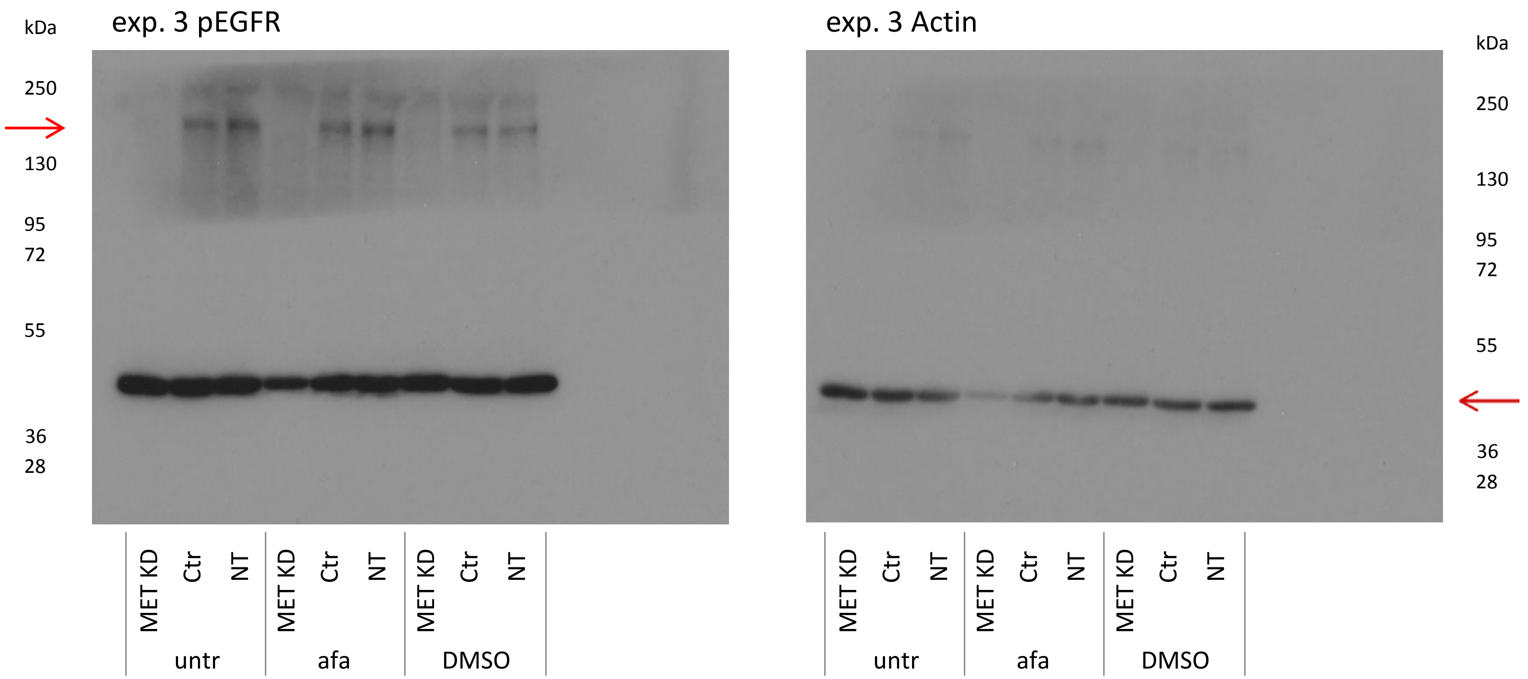
exp. 3 pEGFR
kDa
250
130
95
72
55
36
28
MET KD
Ctr
NT
MET KD
Ctr
NT
MET KD
Ctr
NT
untr
afa
DMSO
exp. 3 Actin
kDa
250
130
95
72
55
36
28
MET KD
Ctr
NT
MET KD
Ctr
NT
MET KD
Ctr
NT
untr
afa
DMSO

Figure 3a,c,e

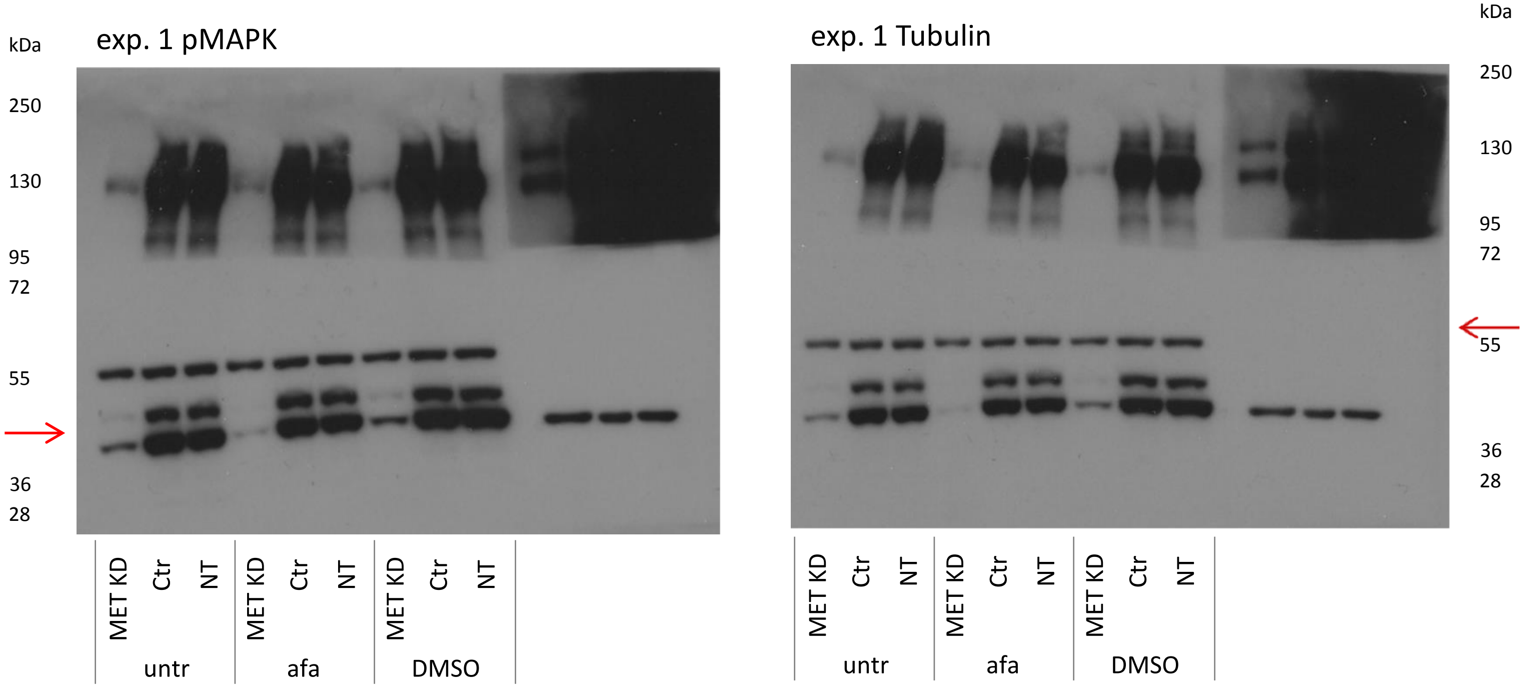
exp. 1 pMAPK
kDa
250
130
95
72
55
36
28
MET KD
Ctr
NT
MET KD
Ctr
NT
MET KD
Ctr
NT
untr
afa
DMSO
exp. 1 Tubulin
kDa
250
130
95
72
55
36
28
MET KD
Ctr
NT
MET KD
Ctr
NT
MET KD
Ctr
NT
untr
afa
DMSO

Figure 3a,c,e

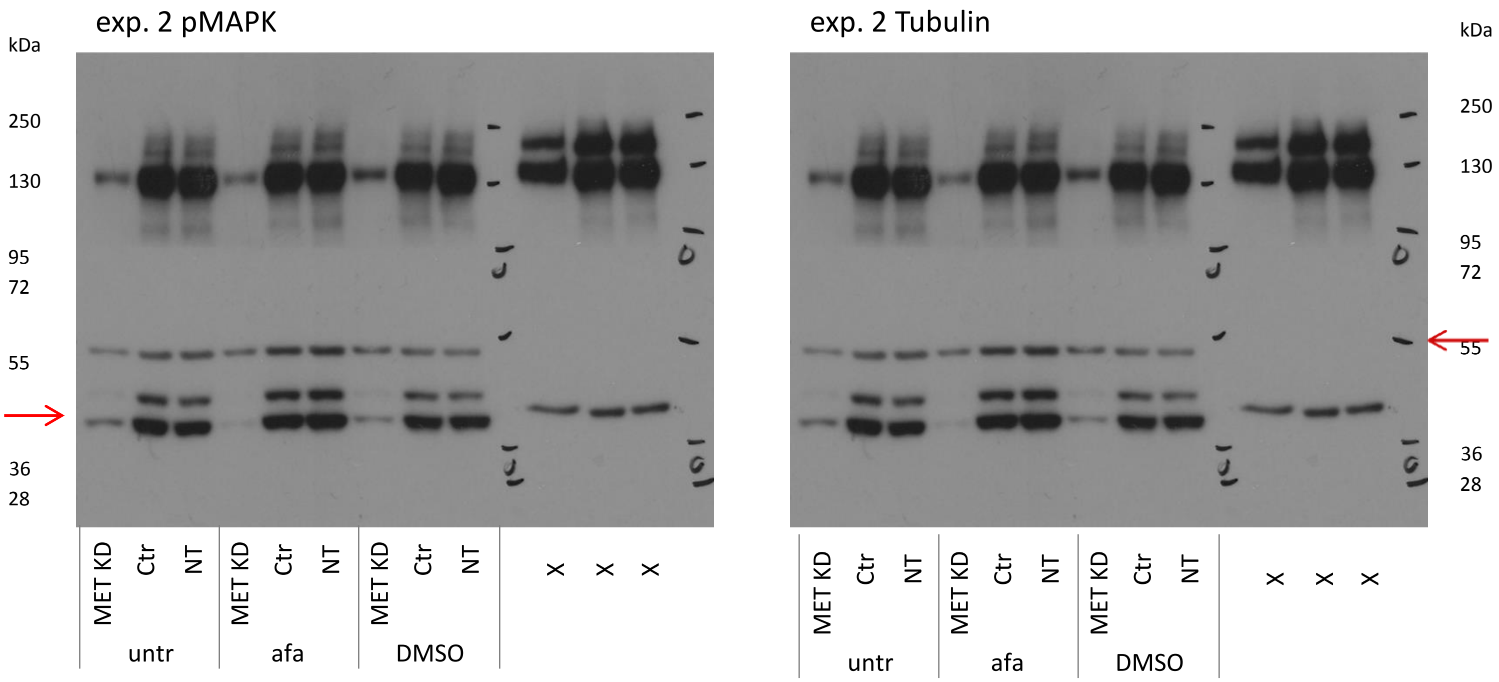
exp. 2 pMAPK
kDa
250
130
95
72
55
36
28
MET KD
Ctr
NT
MET KD
Ctr
NT
MET KD
Ctr
NT
X
X
X
untr
afa
DMSO
exp. 2 Tubulin
kDa
250
130
95
72
55
36
28
MET KD
Ctr
NT
MET KD
Ctr
NT
MET KD
Ctr
NT
X
X
X
untr
afa
DMSO

Figure 3a,c,e

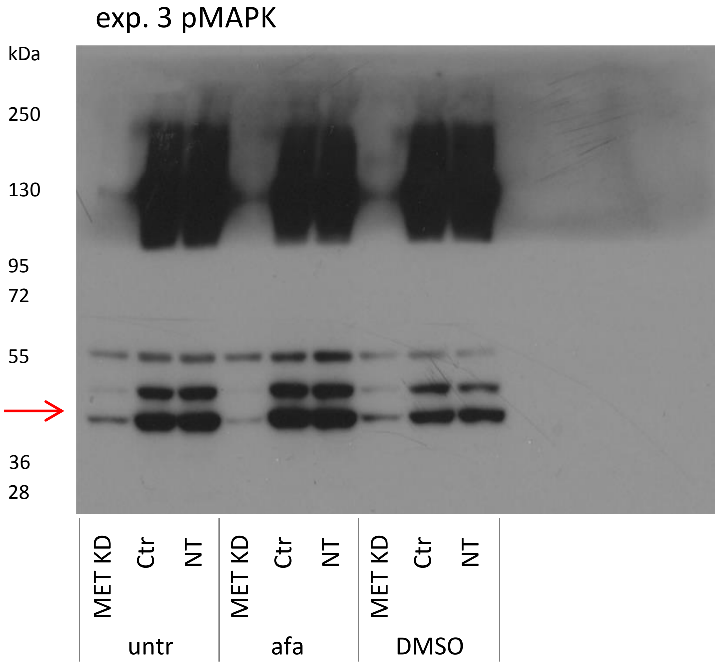
exp. 3 pMAPK
kDa
250
130
95
72
55
36
28
MET KD
Ctr
NT
MET KD
Ctr
NT
MET KD
Ctr
NT
untr
afa
DMSO

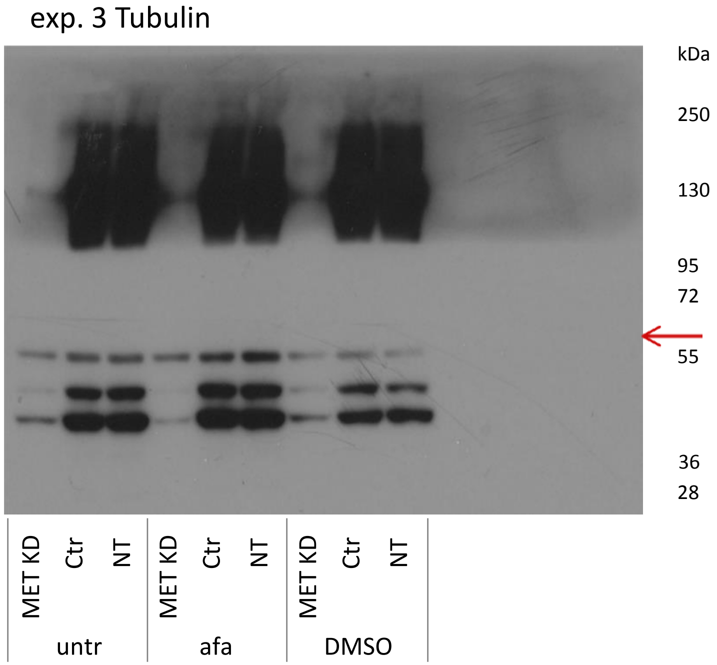
exp. 3 Tubulin
kDa
250
130
95
72
55
36
28
MET KD
Ctr
NT
MET KD
Ctr
NT
MET KD
Ctr
NT
untr
afa
DMSO

Figure 3b,d,f

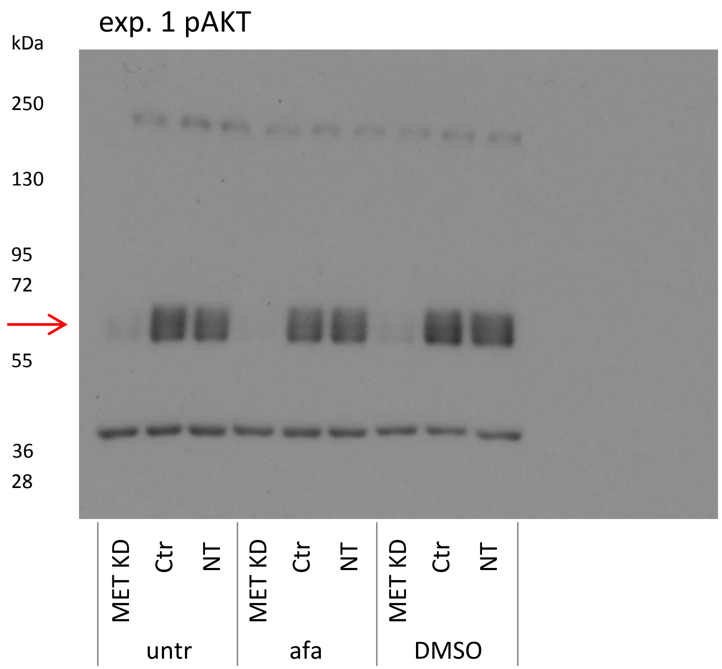
exp. 1 pAKT
kDa
250
130
95
72
55
36
28
MET KD
Ctr
NT
MET KD
Ctr
NT
MET KD
Ctr
NT
untr
afa
DMSO

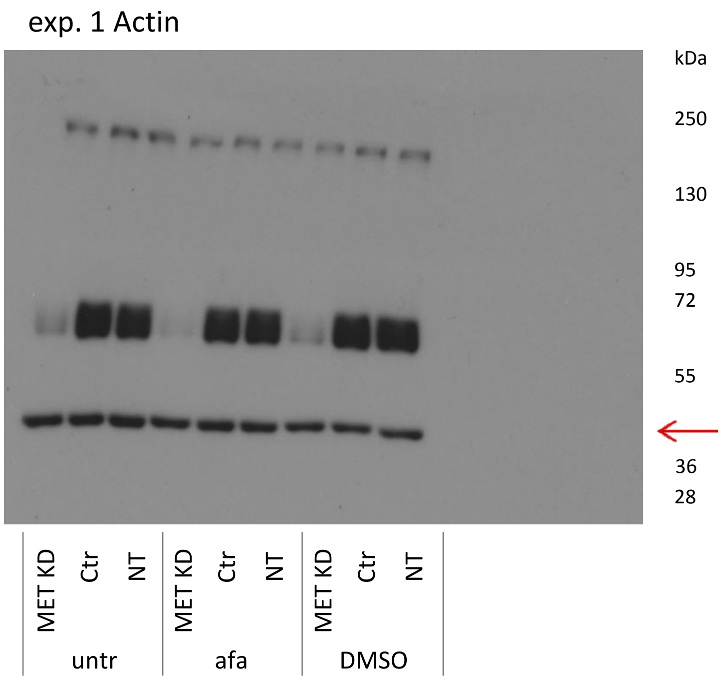
exp. 1 Actin
kDa
250
130
95
72
55
36
28
MET KD
Ctr
NT
MET KD
Ctr
NT
MET KD
Ctr
NT
untr
afa
DMSO

Figure 3b,d,f

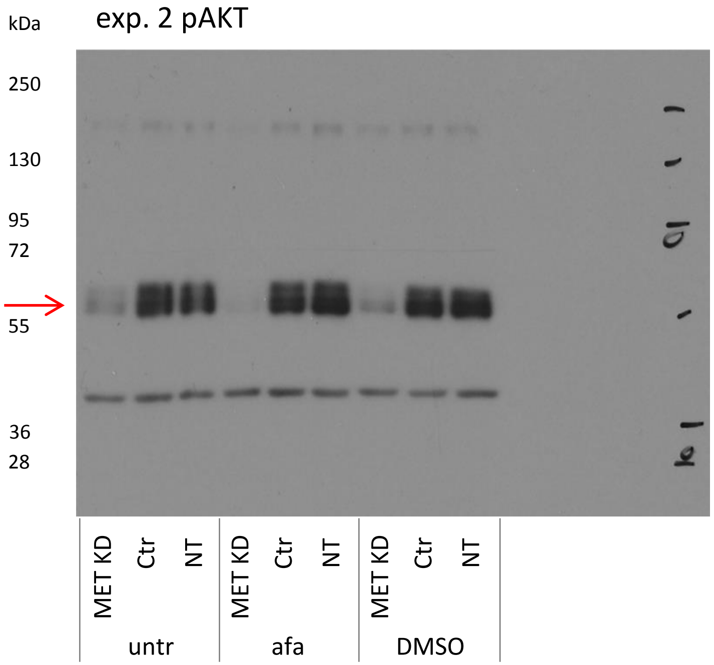
exp. 2 pAKT
kDa
250
130
95
72
55
36
28
MET KD
Ctr
NT
MET KD
Ctr
NT
MET KD
Ctr
NT
untr
afa
DMSO

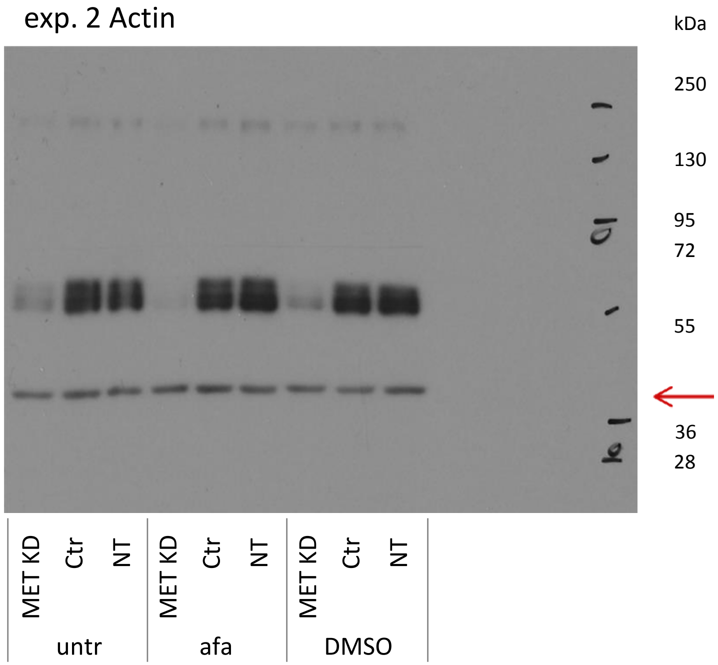
exp. 2 Actin
kDa
250
130
95
72
55
36
28
MET KD
Ctr
NT
MET KD
Ctr
NT
MET KD
Ctr
NT
untr
afa
DMSO

Figure 3b,d,f

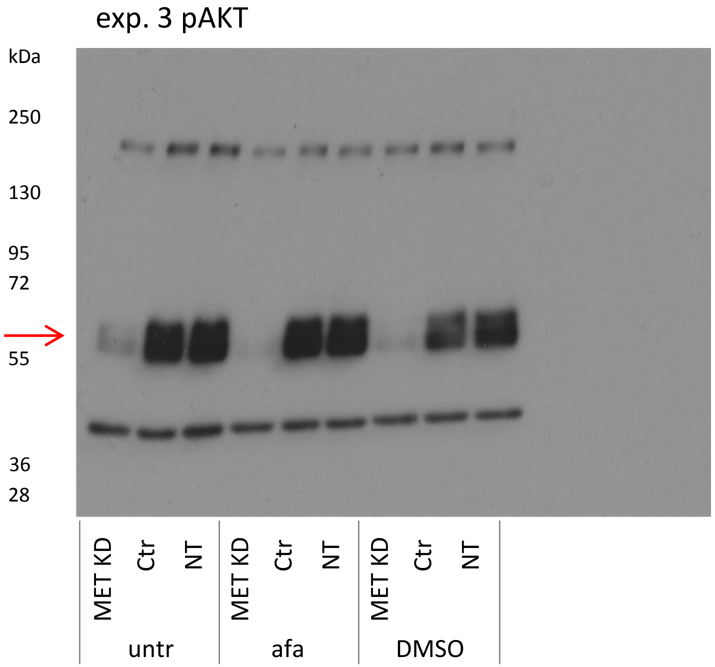
exp. 3 pAKT
kDa
250
130
95
72
55
36
28
MET KD
Ctr
NT
MET KD
Ctr
NT
MET KD
Ctr
NT
untr
afa
DMSO

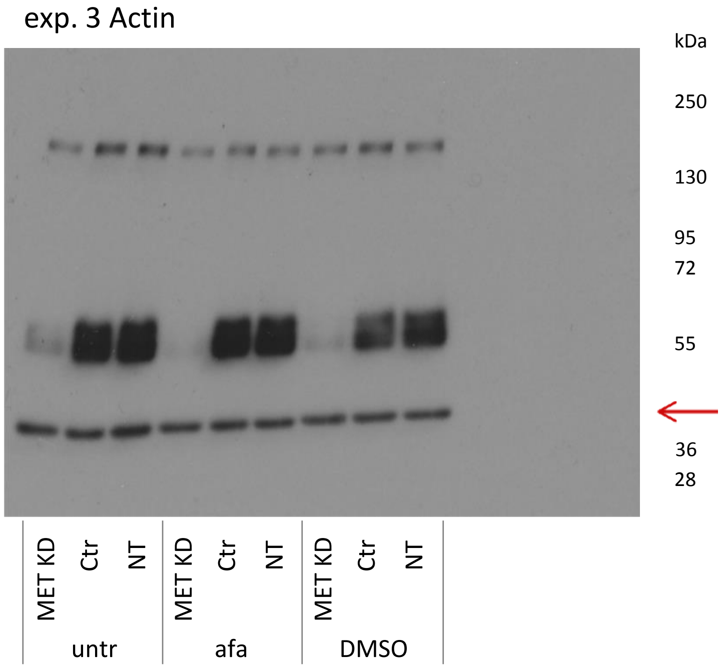
exp. 3 Actin
kDa
250
130
95
72
55
36
28
MET KD
Ctr
NT
MET KD
Ctr
NT
MET KD
Ctr
NT
untr
afa
DMSO
